# Collagen type II suppresses articular chondrocyte hypertrophy and osteoarthritis progression by promoting integrin β1−SMAD1 interaction

**DOI:** 10.1038/s41413-019-0046-y

**Published:** 2019-03-06

**Authors:** Chengjie Lian, Xudong Wang, Xianjian Qiu, Zizhao Wu, Bo Gao, Lei Liu, Guoyan Liang, Hang Zhou, Xiaoming Yang, Yan Peng, Anjing Liang, Caixia Xu, Dongsheng Huang, Peiqiang Su

**Affiliations:** 1 Department of Orthopedics, The First Affiliated Hospital of Sun Yat-sen University, Guangzhou, Guangdong China; 2 Department of Orthopedics, Sun Yat-sen Memorial Hospital of Sun Yat-sen University, Guangzhou, Guangdong China; 3 Department of Orthopedics, The Third Affiliated Hospital of Sun Yat-sen University, Guangzhou, Guangdong China; 4 Department of Microbiology, Zhongshan School of Medicine, Sun Yat-sen University, Guangzhou, Guangdong China; 5 Division of Orthopaedic Surgery, Department of Surgery, Guangdong General Hospital, Guangdong Academy of Medicine Science, Guangzhou, Guangdong China; 6 Research Centre for Translational Medicine, The First Affiliated Hospital of Sun Yat-sen University, Guangzhou, Guangdong China

**Keywords:** Pathogenesis, Bone

## Abstract

Hypertrophic differentiation is not only the terminal process of endochondral ossification in the growth plate but is also an important pathological change in osteoarthritic cartilage. Collagen type II (COL2A1) was previously considered to be only a structural component of the cartilage matrix, but recently, it has been revealed to be an extracellular signaling molecule that can significantly suppress chondrocyte hypertrophy. However, the mechanisms by which COL2A1 regulates hypertrophic differentiation remain unclear. In our study, a *Col2a1* p.Gly1170Ser mutant mouse model was constructed, and Col2a1 loss was demonstrated in homozygotes. Loss of Col2a1 was found to accelerate chondrocyte hypertrophy through the bone morphogenetic protein (BMP)-SMAD1 pathway. Upon interacting with COL2A1, integrin β1 (ITGB1), the major receptor for COL2A1, competed with BMP receptors for binding to SMAD1 and then inhibited SMAD1 activation and nuclear import. COL2A1 could also activate ITGB1-induced ERK1/2 phosphorylation and, through ERK1/2-SMAD1 interaction, it further repressed SMAD1 activation, thus inhibiting BMP-SMAD1-mediated chondrocyte hypertrophy. Moreover, COL2A1 expression was downregulated, while chondrocyte hypertrophic markers and BMP-SMAD1 signaling activity were upregulated in degenerative human articular cartilage. Our study reveals novel mechanisms for the inhibition of chondrocyte hypertrophy by COL2A1 and suggests that the degradation and decrease in COL2A1 might initiate and promote osteoarthritis progression.

## Breaking a destructive cycle

A signaling feedback loop that contributes to cartilage degeneration may offer a fruitful target for the treatment of osteoarthritis. During the early stages of this disorder, cartilage-forming chondrocytes undergo a process of expansion known as hypertrophy, after which they die and are replaced by calcium. Researchers led by Peiqiang Su and Dongsheng Huang of Sun Yat-sen University have demonstrated that COL2A1, an important structural protein, represents an important safeguard against hypertrophy. COL2A1 helps maintain chondrocytes in their normal, healthy state, but Su and Huang showed that signaling factors produced during cartilage repair can reduce COL2A1 levels. This in turn accelerates hypertrophy, promoting further depletion of COL2A1 and ultimately leading to full-blown osteoarthritis. Drugs that break this cycle and preserve COL2A1 could thus help protect endangered joints before the damage becomes severe.

## Introduction

Hypertrophic differentiation of chondrocytes is the terminal stage of endochondral ossification in the growth plate.^1,2^ Hypertrophic differentiated chondrocytes are characterized by an enlarged size, high expression of collagen type X (COL10A1), runt-related transcription factor 2 (RUNX2) and matrix metalloproteinase (MMP), and low expression of cartilaginous specific markers, such as collagen type II (COL2A1) and SRY-box 9 (SOX9). Hypertrophic differentiated chondrocytes eventually undergo apoptosis and are replaced by calcification.^1,2^ However, chondrocyte hypertrophy is also found in degenerative articular cartilage, such as in osteoarthritis (OA). In contrast to endochondral ossification, which involves a programmed process of chondrocyte hypertrophy, healthy mature articular chondrocytes (ACs) remain in a postmitotic quiescent state and resist proliferation and hypertrophy through unknown mechanisms. However, in osteoarthritic cartilage, hypertrophic differentiation, combined with apoptosis and calcification, can be observed in degenerative chondrocytes.^3–5^ Chondrocyte hypertrophy in OA disturbs cartilage homeostasis and is thought to be responsible for OA development.^5,6^ If the underlying mechanisms of the induction of AC hypertrophy can be elucidated, it may be possible to treat OA by inhibiting these pathological changes.^5–7^

COL2A1 is the major component of the cartilage matrix, and together with other proteins and proteoglycans, COL2A1 can form complex extracellular scaffolds to bear mechanical forces, maintain physiological homeostasis, and provide anchoring sites for chondrocytes, extracellular matrix (ECM) molecules, and growth factors.^8^ Degradation and reduction of COL2A1, which are regarded as typical pathological changes, are frequently observed in OA cartilage.^9^ Chondrocyte hypertrophy in OA cartilage is thought to contribute to the COL2A1 decrease.^3,5^ In addition to its structural function, COL2A1 is an important extracellular signaling molecule that can regulate chondrocyte proliferation, metabolism, and differentiation, similar to soluble signals.^10–13^ In this study, Col2a1 loss was demonstrated in a *Col2a1* p.Gly1170Ser mutant mouse, and the decrease in Col2a1 accelerated chondrocyte hypertrophy, indicating that Col2a1 could regulate chondrocyte differentiation as a signaling molecule. However, the specific mechanisms remain unclear.

Integrins are major surface receptors of chondrocytes, and integrin β1 (ITGB1) is considered to be the most common and important receptor for COL2A1.^10,13–15^ In addition to connecting chondrocytes and the ECM, integrins play a vital role in signal transduction, and mitogen-activated protein kinases (MAPKs) act as typical downstream effectors of integrin signaling.^10,13,16,17^ The conditional deletion of *Itgb1* in early limb mesenchyme resulted in accelerated chondrocyte hypertrophy and defects in chondrocyte proliferation,^18^ suggesting that ITGB1 is indispensable for chondrocyte differentiation; however, the molecular mechanisms remain unknown. *Itgb1* gene-deficient mice^18–20^ showed similar cartilage deformities to those with collagen type II alpha1 (*Col2a1*) gene mutations,^21,22^ and in vitro studies have revealed that blockage of ITGB1 can inhibit the influence of COL2A1 on chondrocyte metabolism.^10,13^ However, whether ITGB1 mediates the effect of COL2A1 on chondrocyte hypertrophy deserves further research. Moreover, as reported, the integrin family is involved in OA progression,^23–25^ and integrin α1β1 is able to protect against posttraumatic OA-induced cartilage degradation.^23^ The detailed mechanisms underlying the effect of ITGB1 on OA progression remain unclear.

Transformation growth factor-β (TGFβ)/small mother against decapentaplegic (SMAD) superfamily plays an indispensable role in various biological processes of chondrocytes, including hypertrophy.^7,26–29^ Canonical TGFβ/SMAD signaling can be transduced by the formation of complexes of TGFβ superfamily ligands with type II and I serine/threonine kinase receptors. The binding of ligands allows the constitutively active type II receptor kinases to phosphorylate the dormant type I receptor kinases and then enables the phosphorylation of receptor-regulated SMADs (R-SMADs, including SMAD1/2/3/5/8). R-SMADs bind with common-SMAD (co-SMAD, that is, SMAD4) and translocate into the nucleus to regulate target gene transcription.^26,30^ In addition to canonical signaling pathways, the TGFβ superfamily can extensively interact with many other pathways, and MAPK has been widely reported to be able to phosphorylate the linker region of R-SMADs to inhibit the nuclear accumulation of R-SMADs and reduce their transcriptional activity.^31–35^ The TGFβ/SMAD superfamily can be divided into two branches: the TGFβ-SMAD2/3 branch and the BMP-SMAD1/5/8 branch. BMP-SMAD1/5/8 has a stimulatory effect on chondrocyte hypertrophy, while TGFβ-SMAD2/3 has the opposite effect.^26^ The TGFβ and BMP pathways collaborate in regulating the differentiation state of chondrocytes and maintaining cartilage homeostasis.^26,36^ During OA, the balance of TGFβ/BMP signaling is disturbed and contributes to AC hypertrophy and OA development.^7,26,29,36,37^ However, the detailed mechanism underlying the imbalance between TGFβ and BMP activity in OA cartilage remains unclear.

The current study focused on the signaling function of COL2A1 and demonstrated that COL2A1 inhibited chondrocyte hypertrophy and OA progression via the suppression of BMP-SMAD1 pathway activity. Furthermore, the specific mechanisms by which COL2A1 influences BMP-SMAD1 signaling are illustrated.

## Results

### Chondrocytes from *Col2a1* mutant mice underwent enhanced hypertrophic differentiation due to Col2a1 loss

The generation of *Col2a1* p.Gly1170Ser knockin mice and the detailed phenotypes of all genotypes, that is, wild types (WT), heterozygotes (Hetero), and homozygotes (Homo), have been previously described.^21^

**Fig. 1:**
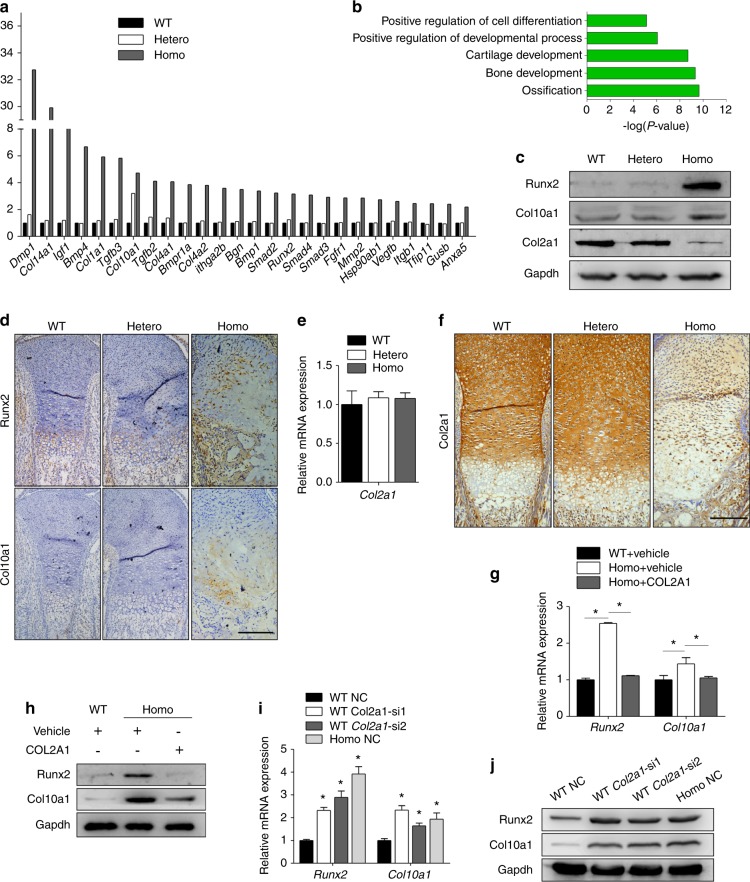
Chondrocytes from *Col2a1* mutant mice underwent enhanced hypertrophic differentiation due to Col2a1 loss. **a** *Col2a1* p.Gly1170Ser knockin mice were constructed, and the primary chondrocytes isolated from the articular cartilage of embryos of all three genotypes were cultured for 7 d. Then, we performed a qPCR array analysis (the experiments were replicated with four different litters of embryos). Differentially expressed genes (*P* *<* 0.05, with a fold change of >2 or a fold change of <0.5) in homozygotes are shown in the histogram. **b** Gene ontology annotation analysis of the biological processes of the differentially expressed genes from the qPCR array assay. Enrichment scores corresponding to each pathway provided by the DAVID annotation tool are displayed as −log(*P* values). A term was considered to be significantly enriched only if it passed the count threshold of six genes per annotation term and presented an EASE score, with the Benjamini−Hochberg correction set to 0.05. In the DAVID database, the EASE score is a modified Fisher exact *P* value used for enrichment analysis within gene lists, with *P* value = 0 representing perfect enrichment. **c** Immunoblotting analysis was performed to detect the protein levels of Runx2, Col10a1, and Col2a1 in chondrocytes of all three genotypes. **d** Representative examples of IHC staining of Runx2 and Col10a1 in the growth plates of the three genotypes. Scale bars: 200 μm. **e**, **f** Col2a1 expression was detected by qPCR (**e**) and IHC (**f**) in all three genotypes. Scale bars: 200 μm. **g**, **h** Wild-type chondrocytes were treated with vehicle (0.05 M acetic acid), while homozygous chondrocytes were treated with COL2A1 (100 μg·mL^−1^) or vehicle for 48 h. Runx2 and Col10a1 expression levels were detected by qPCR (**g**) and immunoblotting (**h**). **i**, **j** After silencing *Col2a1* by siRNA in wild-type chondrocytes, the expression levels of Runx2 and Col10a1 were detected by qPCR (**i**) and immunoblotting (**j**). Data in (**e**), (**g**), and (**i**) are presented as the mean ± SD (*n* = 3). *P < 0.05. IHC, immunohistochemistry

To evaluate the influence of the *Col2a1* mutation on endochondral ossification-related pathways, a qPCR analysis was conducted to profile the expression of 84 pathway genes in chondrocytes from all genotypes (the complete data are provided in Supplementary Table 1 and Supplementary Fig. 1a). Differentially expressed genes are listed in Fig. 1a and divided into four groups in Supplementary Table 2. There were no significant differences between the wild types and the heterozygotes, but 26 genes were upregulated in the homozygotes. These 26 genes were submitted to gene ontology annotations of biological processes to determine the most significantly overrepresented biological process. After manually removing redundant terms, “ossification”, “bone development”, “cartilage development”, “positive regulation of developmental process”, and “positive regulation of cell differentiation” were identified (Fig. 1b). Together with the significantly upregulated hypertrophic markers of *Col10a1*, *Col1a1*, *Runx2*, and *Dmp1* (Supplementary Table 2), these alterations indicated accelerated chondrocyte hypertrophy. Immunoblotting confirmed that the expression levels of Runx2 and Col10a1 were upregulated in homozygotes, while no obvious difference was found between wild types and heterozygotes (Fig. 1c). Immunohistochemical (IHC) staining showed that the normal architecture of the growth plate disappeared in homozygotes, while the heterozygous growth plate remained almost normal. In the homozygous growth plate, the regular alignment of proliferative and hypertrophic cells was disturbed. Resting and hypertrophic-like chondrocytes could still be found, while proliferating chondrocytes became fusiform and almost disappeared. The expression levels of Runx2 and Col10a1 were much higher in homozygotes, especially in hypertrophic-like chondrocytes (Fig. 1d).

The *Col2a1* p.Gly1170Ser mutation can cause an endoplasmic reticulum stress (ERS)-unfolding protein reaction (UPR)-apoptosis cascade.^21^ In homozygotes, misfolded Col2a1 is retained and degraded in the ER, leading to Col2a1 loss. qPCR demonstrated no significant difference in *Col2a1* mRNA levels among the three genotypes (Fig. 1e). Immunoblotting and IHC assays showed a sharply reduced Col2a1 protein level in homozygotes, while there was no obvious difference between wild types and heterozygotes (Fig. 1c, f). Transmission electron microscopy analysis revealed an obvious reduction in collagen fibers in the homozygous cartilage matrix throughout the entire growth plate (Supplementary Fig. 1b). The above data showed decreased Col2a1 protein in homozygotes, which was caused by its degradation.

To verify that the loss of Col2a1 caused enhanced hypertrophy, a rescue experiment was performed in which purified COL2A1 was added to a culture of homozygous chondrocytes. COL2A1 supplementation partially downregulated the expression of hypertrophic markers, which was highly elevated in homozygotes (Fig. 1g, h). *Col2a1* silencing upregulated hypertrophic markers to a level similar to that found in the homozygotes (Fig. 1i, j; Supplementary Fig. 1c, d).

### COL2A1 exerted suppression on chondrocyte hypertrophy and on the production of matrix-degrading enzymes

COL2A1 expression in SW1353 (a human chondrosarcoma cell line), Hs819.T (a human chondrosarcoma cell line), and 293T cells was much lower than that in human ACs, while Col2a1 expression in ATDC5 cells (a mouse chondrogenic cell line) was similar to that found in mouse primary ACs (Supplementary Fig. 2a−d).

**Fig. 2:**
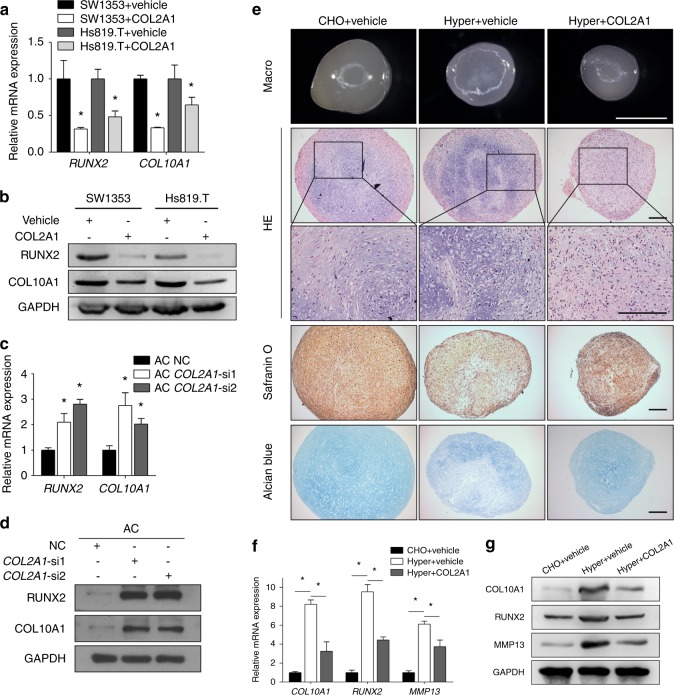
COL2A1 exerted suppression on chondrocyte hypertrophy and on the production of matrix-degrading enzymes. **a**, **b** SW1353 and Hs819.T cells were treated with COL2A1 (100 μg·mL^−1^) or vehicle (0.05 mol·L^−1^ acetic acid) for 48 h, and the expression levels of RUNX2 and COL10A1 were detected by qPCR (**a**) and immunoblotting (**b**). **c**, **d** Human articular chondrocytes were transfected with *COL2A1* siRNA or negative control siRNA, and the expression levels of RUNX2 and COL10A1 were detected by qPCR (**c**) and immunoblotting (**d**). **e**−**g** Pellet cultures of MSCs were induced to undergo chondrogenesis for 14 d and then induced for hypertrophic differentiation for another 14 d with purified COL2A1 or vehicle. In addition, pellet cultures of MSCs induced to undergo chondrogenesis for 28 d were used as chondrogenic controls. **e** Gross appearance, HE staining, safranin O staining, and Alcian blue staining were evaluated. Scale bars in the images of gross appearance: 1 mm; other scale bars in (**e**): 200 μm. The expression levels of COL10A1, RUNX2, and MMP13 were detected by qPCR (**f**) and immunoblotting (**g**). Data in (**a**), (**c**), and (**f**) are presented as the mean ± SD (*n* = 3). *P < 0.05. AC, articular chondrocyte; CHO, chondrogenic differentiation; Hyper, hypertrophic differentiation; MSC, mesenchymal stem cell

SW1353 and Hs819.T cells underwent an additional COL2A1 treatment,^10^ and the treatment suppressed both mRNA and protein expression of the hypertrophic markers (Fig. 2a, b). A TUNEL assay was then conducted to indicate the late hypertrophic status (i.e., apoptosis). Cells were treated with IL-1β to both induce OA-like pathological changes and increase the apoptotic rate,^38,39^ and simultaneous COL2A1 stimulation reduced apoptotic rates (Supplementary Fig. 2e). Silencing *COL2A1* (or *Col2a1*) not only upregulated the expression of hypertrophic markers (Fig. 2c, d; Supplementary Fig. 2f-k) but also increased the percentage of apoptotic cells (Supplementary Fig. 2l, m).

Furthermore, the effect of COL2A1 on the hypertrophy of chondrifying mesenchymal stem cells (MSCs) was detected. Pellet cultures of human MSCs were first induced to undergo chondrogenesis for 14 d and then induced to undergo hypertrophic differentiation for another 14 d with purified COL2A1 or vehicle. Additional treatment with COL2A1 did not significantly influence the size of the cartilage pellets (Fig. 2e). In histological studies, the COL2A1 group exhibited a more homogeneous hyaline cartilage-like morphology, with fewer hypertrophic chondrocytes (Fig. 2e). Moreover, qPCR and immunoblotting assays confirmed that, in the COL2A1 group, the expression of hypertrophic markers was significantly downregulated (Fig. 2f, g).

The effect of COL2A1 on the production of matrix-degrading enzymes was assessed. COL2A1 treatment in human ACs downregulated the expression of *MMP9*, *MMP13*, *ADAMTS4*, and *ADAMTS5*, while *COL2A1* silencing exerted the opposite effect (Supplementary Fig. 2n, o), suggesting that COL2A1 not only inhibited chondrocyte hypertrophy but also suppressed the production of catabolic enzymes.

### COL2A1 suppressed chondrocyte hypertrophy through regulation of the BMP-SMAD1 pathway

**Fig. 3:**
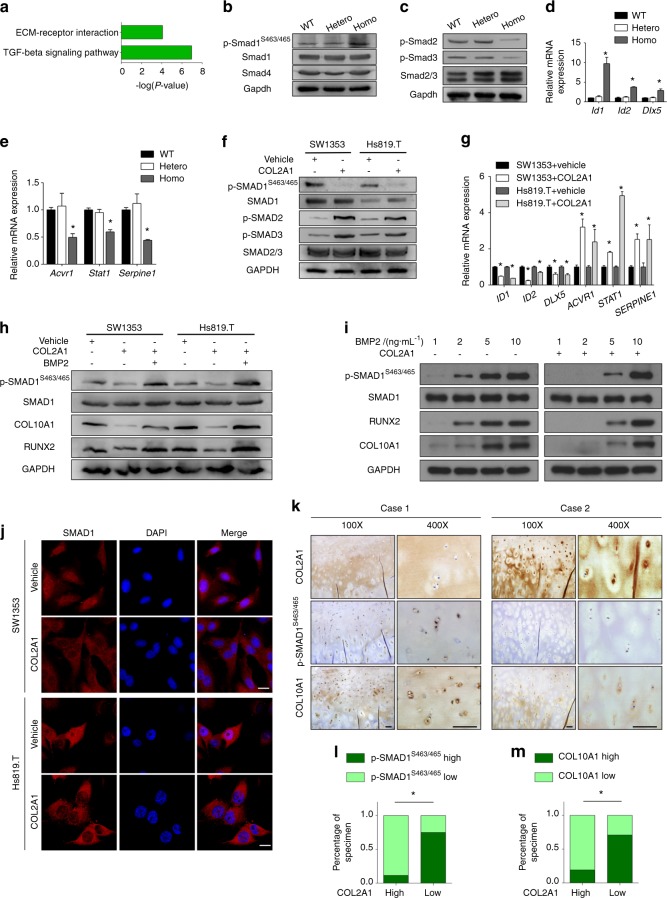
COL2A1 suppressed chondrocyte hypertrophy through regulation of the BMP-SMAD1 pathway. **a** KEGG pathway functional annotations of the differentially expressed genes according to the qPCR analysis. Enrichment scores are displayed as −log(*P* value). A pathway was considered to be significantly enriched only if it passed the count threshold of five genes per annotation term and presented an EASE score, with the Benjamini−Hochberg correction set to 0.05. **b**, **c** Immunoblotting evaluation of p-Smad1^S463/465^, Smad1, Smad4, p-Smad2, p-Smad3, and Smad2/3 in chondrocytes of all three genotypes from *Col2a1* p.Gly1170Ser mutant mice. **d**, **e** qPCR quantification of *Id1*, *Id2*, *Dlx5*, *Acvr1*, *Stat1*, and *Serpine1* in chondrocytes of *Col2a1* mutant mice. **f**, **g** Immunoblotting evaluation of p-SMAD1^S463/465^, p-SMAD2, p-SMAD3, SMAD1, and SMAD2/3 (**f**), and qPCR quantification of *ID1*, *ID2*, *DLX5*, *ACVR1*, *STAT1*, and *SERPINE1* (**g**) were carried out in SW1353 and Hs819.T cells treated with 100 μg·mL^−1^ purified COL2A1 or vehicle (0.05 mol·L^−1^ acetic acid). **h** Immunoblotting evaluation of p-SMAD1^S463/465^, SMAD1, RUNX2, and COL10A1 in SW1353 and Hs819.T cells treated with vehicle (0.05 mol·L^−1^ acetic acid), 100 μg·mL^−1^ COL2A1, or 10 ng·mL^−1^ BMP2. **i** Human articular chondrocytes were treated with the indicated concentrations of BMP2 and 100 μg·mL^−1^ purified COL2A1, and the expression levels of p-SMAD1^S463/465^, SMAD1, RUNX2, and COL10A1 were detected by immunoblotting. **j** Confocal laser scanning was used to demonstrate SMAD1 nuclear localization in SW1353 and Hs819.T cells treated with COL2A1 or vehicle. Scale bars: 20 μm. **k**−**m** Injured cartilage samples from 50 OA patients underwent immunohistochemical staining using anti-COL2A1, p-SMAD1^S463/465^, and COL10A1 antibodies. **k** Representative micrographs of two patients are shown. Scale bars: 100 μm. **l**, **m** The correlation among the expression levels of three proteins was analyzed by the chi-squared test. Data in (**d**), (**e**), and (**g**) are presented as the mean ± SD (*n* = 3). *P < 0.05

To determine which signaling pathways are involved in the transduction of COL2A1’s effect on chondrocyte hypertrophy, the differentially expressed genes obtained from the qPCR array were submitted to functional annotations from Kyoto Encyclopedia of Genes and Genomes (KEGG) pathway analysis. After manually removing the redundant terms, “TGFβ signaling pathway” and “ECM−receptor interaction” were considered to be significantly enriched (Fig. 3a). Considering the majority of differentially expressed genes belonging to the TGFβ/BMP superfamily (Supplementary Table 2), the activities of both TGFβ-SMAD2/3 and BMP-SMAD1/5/8 branches were examined in *Col2a1* mutant mouse chondrocytes. Similar to the qPCR array analysis, no obvious differences were found between heterozygotes and wild types. However, in homozygotes, Smad1 phosphorylation (Ser463/Ser465) and its typical early-response genes (*Id1*, *Id2*, and *Dlx5*) were upregulated, while p-Smad2/3 and their typical early-response genes (*Acvr1*, *Stat1*, and *Serpine1*) were slightly downregulated (Fig. 3b−e). Additional COL2A1 treatment downregulated p-SMAD1^S463/465^ and its typical early-response genes but upregulated the phosphorylation of both SMAD2 and SMAD3 and their typical early-response genes (Fig. 3f, g). Silencing *COL2A1* (or *Col2a1*) induced the opposite effect (Supplementary Fig. 3a-d).

Whether BMP-SMAD1 or TGFβ-SMAD2/3 mediated the effect of COL2A1 was also studied. High-dose BMP2 treatment, which activated SMAD1, was demonstrated to be able to release the inhibitory effect of COL2A1 on chondrocyte hypertrophy (Fig. 3h). In addition, COL2A1 was shown to be able to suppress BMP2-induced chondrocyte hypertrophy (Fig. 3i), suggesting that COL2A1 inhibited chondrocyte hypertrophy through BMP-SMAD1 signaling. However, SB505124, which blocked SMAD2/3 activation, could not reverse the repression of COL2A1 on chondrocyte hypertrophy (Supplementary Fig. 3e). Confocal laser scanning showed that COL2A1 treatment inhibited the nuclear import of SMAD1 (Fig. 3j), while *Col2a1* silencing facilitated this process (Supplementary Fig. 3f).

To confirm the clinical relevance and validity of the current study, the expression of COL2A1, p-SMAD1^S463/465^, and COL10A1 and their correlations were examined in cartilage samples derived from 50 patients with knee OA. IHC and semiquantitative assays were performed. The specimens were divided into high COL2A1 protein and low COL2A1 protein expression groups based on the median as a cutoff value. The COL2A1 high expression group was composed of 7 males and 19 females with a mean age of (68.04 ± 8.41) years. The COL2A1 low expression group was composed of 7 males and 17 females with a mean age of (67.38 ± 6.83) years. The gender and age distributions were not significantly different between the two groups. Lower expression levels of p-SMAD1^S463/465^ and COL10A1 were found in the COL2A1 high expression group (Fig. 3k−m).

### ITGB1 receptor mediated the effect of COL2A1 on the BMP pathway and chondrocyte hypertrophy

**Fig. 4:**
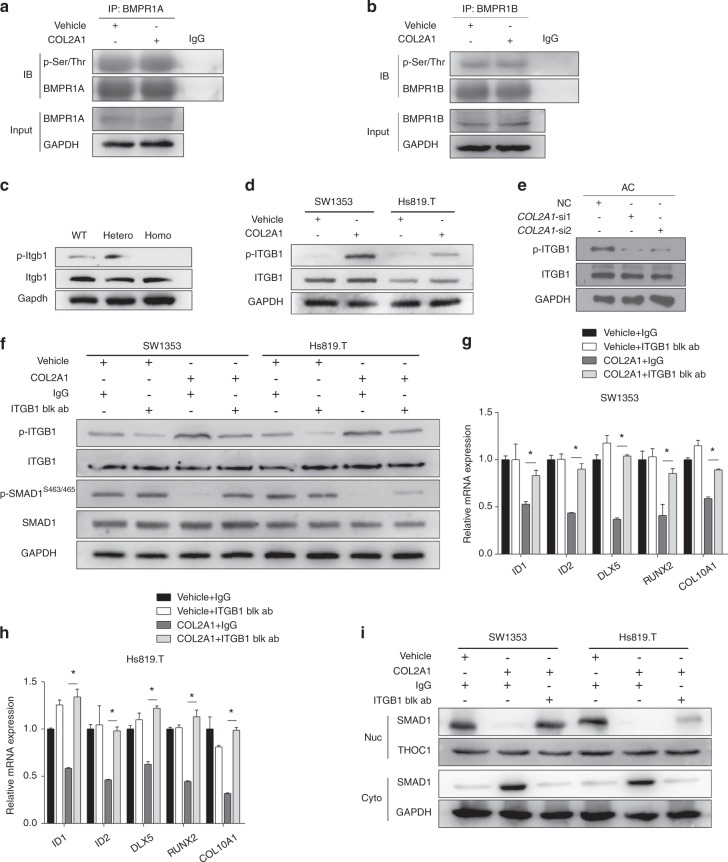
The ITGB1 receptor mediated the effect of COL2A1 on the BMP pathway and chondrocyte hypertrophy. **a**, **b** Immunoprecipitation with anti-BMPR1A (**a**) or anti-BMPR1B (**b**) antibody was performed in extracts of 293T cells treated with 100 μg·mL^−1^ COL2A1 or vehicle (0.05 mol·L^−1^ acetic acid) for 1 h, and then, immunoblotting was conducted with anti-phospho-serine/threonine and anti-BMPR1A/B antibodies. **c**−**e** Immunoblotting evaluation of p-ITGB1 and ITGB1 in chondrocytes of *Col2a1* p.Gly1170Ser mutant mice (**c**), SW1353 and Hs819.T cells treated with COL2A1 or vehicle (**d**), and human articular chondrocytes transfected with *COL2A1* siRNAs or negative control siRNA (**e**). **f**−**h** SW1353 and Hs819.T cells were pretreated with 10 μg·mL^−1^ ITGB1 blocking antibody or anti-human IgG antibody for 1 h and then treated with COL2A1 or vehicle for 1 h. Immunoblotting evaluation of p-ITGB1, p-SMAD1^S463/465^, ITGB1, and SMAD1 (**f**), and qPCR evaluation of *ID1*, *ID2*, *DLX5*, *RUNX2*, and *COL10A1* (**g, h**) were performed. **i** Immunoblotting evaluation of SMAD1 in both nuclear (Nuc) and cytoplasmic (Cyto) extracts was performed in SW1353 and Hs819.T cells pretreated with ITGB1 blocking antibody or anti-human IgG and then treated with COL2A1 or vehicle. Data in (**g**) and (**h**) are presented as the mean ± SD (*n* = 3). *P < 0.05. AC, articular chondrocyte; Blk ab, blocking antibody

To determine how COL2A1 represses BMP-SMAD1 signaling, the ability of COL2A1 to directly act on bone morphogenetic protein receptors (BMPRs) was first studied. Serine/threonine phosphorylation of BMPR1A and BMPR1B was assessed to indicate the activity of the BMP receptor complex. There were no observable differences in the phosphorylation of either receptor, regardless of COL2A1 treatment (Fig. 4a, b), suggesting that COL2A1 has no direct effect on BMPRs.

The effect of the ITGB1 receptor in mediating COL2A1 signaling was then studied. Itgb1 phosphorylation was significantly reduced in *Col2a1* mutant homozygotes (Fig. 4c). SW1353 and Hs819.T cells treated with COL2A1 showed an obvious increase in ITGB1 phosphorylation (Fig. 4d), while *COL2A1* (or *Col2a1*) silencing resulted in significant downregulation of p-ITGB1 (Fig. 4e and Supplementary Fig. 4). COL2A1 treatment increased ITGB1 phosphorylation and decreased SMAD1^S463/465^ phosphorylation. However, when ITGB1 was blocked, the influence of COL2A1 was counterbalanced (Fig. 4f). In addition, the effect of COL2A1 on BMP-SMAD1 target genes and hypertrophic markers was also reversed by blocking ITGB1 (Fig. 4g, h). Moreover, the nuclear import of SMAD1 was shown to be inhibited by COL2A1 stimulation, while blocking ITGB1 reversed the effects of COL2A1 (Fig. 4i).

### COL2A1 repressed BMP-SMAD1 signaling activation by facilitating ITGB1−SMAD1 interaction and weakening BMPR1A/B−SMAD1 interaction

**Fig. 5:**
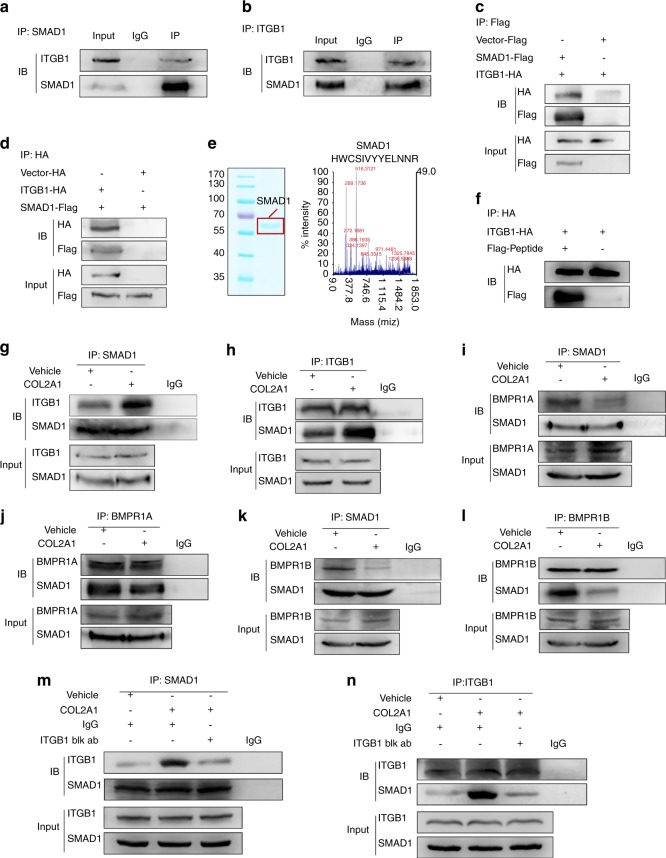
COL2A1 repressed BMP-SMAD1 signaling activation through facilitating the ITGB1−SMAD1 interaction and weakening the BMPR1A/B−SMAD1 interaction. **a**, **b** Immunoprecipitation was performed on 293T extracts with anti-SMAD1 antibody or anti-ITGB1 antibody, followed by immunoblotting with anti-SMAD1 antibody and anti-ITGB1 antibody. **c** 293T cells were transfected with SMAD1-Flag, ITGB1-HA or vector-Flag plasmids, and immunoprecipitation was carried out with anti-Flag antibody followed by immunoblotting with anti-HA antibody and anti-Flag antibody. **d** 293T cells were transfected with SMAD1-Flag, ITGB1-HA, or vector-HA plasmids, and immunoprecipitation was carried out with anti-HA antibody, followed by immunoblotting with anti-HA antibody and anti-Flag antibody. **e** SMAD1 protein was purified from 293T cell extracts expressing SMAD1-Flag and identified by Coomassie blue staining (left panel) and mass spectrometry (right panel). **f** Purified SMAD1-Flag protein was incubated with the product expressing ITGB1-HA, which had been linked to HA affinity agarose beads for 4 h at 4 °C, followed by bead washing and immunoblotting. **g**, **h** Immunoprecipitation was performed on 293T cells treated with 100 μg·mL^−1^ COL2A1 or vehicle (0.05 mol·L^−1^ acetic acid) for 1 h with anti-SMAD1 antibody (**g**) or anti-ITGB1 antibody (**h**), followed by immunoblotting with anti-SMAD1 antibody and anti-ITGB1 antibody. **i**, **j** Immunoprecipitation was performed on 293T cells treated with COL2A1 or vehicle with anti-SMAD1 antibody (**i**) or anti-BMPR1A antibody (**j**), followed by immunoblotting with anti-SMAD1 antibody and anti-BMPR1A antibody. **k**, **l** Immunoprecipitation was performed with anti-SMAD1 antibody (**k**) or anti-BMPR1B antibody (**l**) on 293T cells treated with COL2A1 or vehicle followed by immunoblotting with anti-SMAD1 antibody and anti-BMPR1B antibody. **m**, **n** 293T cells were pretreated with 10 μg·mL^−1^ ITGB1 blocking antibody or anti-human IgG antibody for 1 h and then treated with COL2A1 or vehicle for 1 h. Immunoprecipitation with anti-SMAD1 antibody (**m**) or anti-ITGB1 antibody (**n**) was conducted, followed by immunoblotting with anti-SMAD1 antibody and anti-ITGB1 antibody. Blk ab, blocking antibody

Because COL2A1 was shown to suppress BMP-SMAD1 activity via ITGB1, we examined whether ITGB1 could interact directly with SMAD1. 293T cells were treated with purified COL2A1, and both exogenous and endogenous reciprocal coimmunoprecipitations were performed to identify the ITGB1−SMAD1 interaction (Fig. 5a−d). Flag-tagged SMAD1 was expressed in 293T cells, and the cellular extracts were used for purifying SMAD1 protein. Purified SMAD1 protein was subsequently identified by Coomassie blue staining and mass spectrometry (Fig. 5e). IP and immunoblotting assays were then performed to affirm that SMAD1 is a binding partner of ITGB1 (Fig. 5f). Furthermore, COL2A1 treatment was demonstrated to be able to enhance ITGB1−SMAD1 interaction, while *COL2A1* silencing suppressed their binding (Fig. 5g, h; Supplementary Fig. 5a). COL2A1 stimulation impaired the binding between SMAD1 and BMPR1A/B (Fig. 5i−l), and ITGB1 blockage reversed COL2A1’s promotion of the ITGB1−SMAD1 interaction (Fig. 5m, n). Finally, COL2A1 treatment released BMP2-inhibited ITGB1−SMAD1 interaction (Supplementary Fig. 5b), while BMP2 treatment suppressed the promotion of COL2A1 on the ITGB1−SMAD1 interaction (Supplementary Fig. 5c), indicating that COL2A1 could also regulate the ITGB1−SMAD1 interaction under BMP2 stimulation.

### COL2A1 phosphorylated SMAD1^S206^ by activating ERK1/2, which exerted a negative influence on BMP-SMAD1 activity

**Fig. 6:**
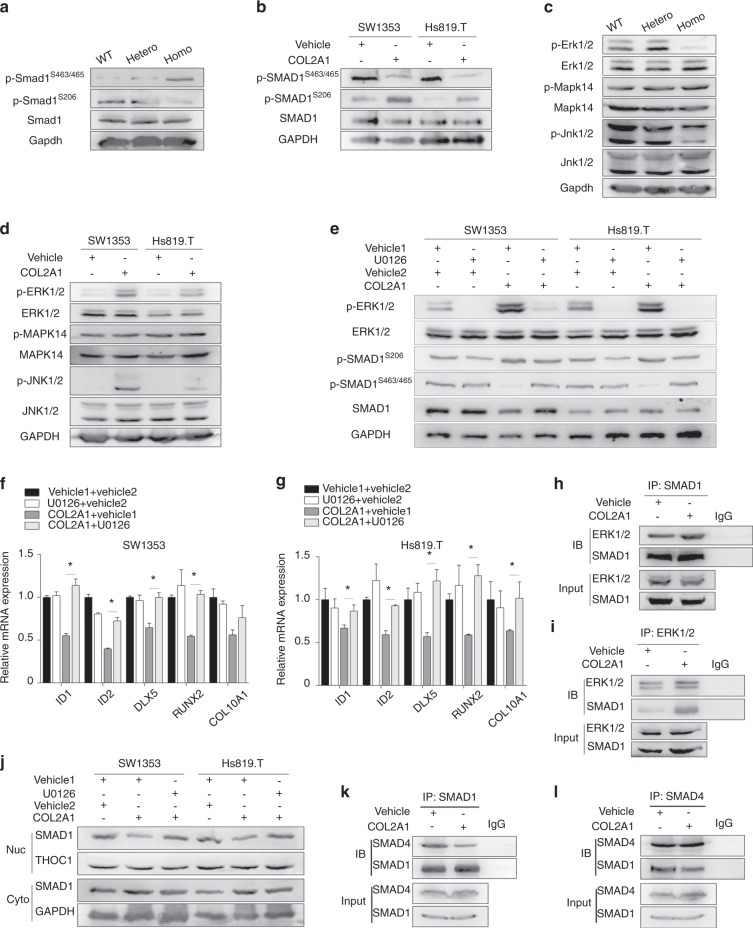
COL2A1 phosphorylated SMAD1^S206^ by activating ERK1/2, which negatively influenced BMP-SMAD1 activity. **a**, **b** Immunoblotting evaluation of p-SMAD1^S463/465^, p-SMAD1^S206^, and SMAD1 in chondrocytes from *Col2a1* mutant mice (**a**) and SW1353 and Hs819.T cells treated with 100 μg·mL^−1^ COL2A1 or vehicle (0.05 mol·L^−1^ acetic acid) for 1 h (**b**). **c**, **d** Immunoblotting evaluation of p-ERK1/2, ERK1/2, p-MAPK14, MAPK14, p-JNK1/2, and JNK1/2 in chondrocytes from *Col2a1* mutant mice (**c**), and SW1353 and Hs819.T cells treated with COL2A1 or vehicle (**d**). **e**−**g** The expression levels of p-ERK1/2, p-SMAD1^S463/465^, p-SMAD1^S206^, ERK1/2, and SMAD1 proteins were detected by immunoblotting (**e**), and the expression levels of *ID1*, *ID2*, *DLX5*, *RUNX2*, and *COL10A1* were tested by qPCR in SW1353 (**f**) and Hs819.T cells (**g**) pretreated with 10 μmol·L^−1^ U0126 or vehicle 1 (dimethylsulfoxide) for 1 h and then treated with 100 μg·mL^−1^ COL2A1 or vehicle 2 (0.05 mol·L^−1^ acetic acid) for 1 h. **h**, **i** Immunoprecipitation was performed on 293T cells treated with COL2A1 or vehicle with anti-SMAD1 antibody (**h**) or anti-ERK1/2 antibody (**i**), followed by immunoblotting with anti-ERK1/2 and anti-SMAD1 antibodies. **j** Immunoblotting evaluation of SMAD1 in both nuclear (Nuc) and cytoplasmic (Cyto) extracts in SW1353 and Hs819.T cells pretreated with U0126 or vehicle 1 and then treated with COL2A1 or vehicle 2. **k, l** Immunoprecipitation was performed on 293T cells treated with COL2A1 or vehicle with anti-SMAD1 antibody (**k**) or anti-SMAD4 antibody (**l**), followed by immunoblotting with anti-SMAD4 antibody and anti-SMAD1 antibody. Data in (**f**) and (**g**) are presented as the mean ± SD (*n* = 3). *P < 0.05

Contrary to SMAD1^S463/465^ phosphorylation, SMAD1^S206^ (SMAD1 linker region) phosphorylation can downregulate SMAD1 transcriptional activity.^31,33^ In this study, Smad1^S206^ phosphorylation was found to decrease in *Col2a1* mutant homozygous chondrocytes (Fig. 6a). In SW1353 and Hs819.T cells, COL2A1 stimulation promoted SMAD1^S206^ phosphorylation (Fig. 6b). MAPKs, including ERK1/2, JNK1/2, and MAPK14, are major protein kinases that can phosphorylate SMAD1^S206^.^31^ Therefore, whether MAPKs mediated the effect of COL2A1 was studied. The phosphorylation of Erk1/2 and Jnk1/2 was decreased in *Col2a1* homozygous mutant chondrocytes, while there was no difference in Mapk14 phosphorylation (Fig. 6c). Moreover, in SW1353 and Hs819.T cells, COL2A1 treatment increased p-ERK1/2 and p-JNK1/2 expression, while it had no effect on MAPK14 phosphorylation (Fig. 6d). In brief, ERK1/2 and JNK1/2 responded to COL2A1 stimulation.

Whether ERK1/2 or JNK1/2 mediated the impact of COL2A1 was studied. U0126, which blocked ERK1/2 phosphorylation, can abrogate the effect of COL2A1 on SMAD1 phosphorylation and the expression of hypertrophic markers to some extent (Fig. 6e−g). Instead, SP600125, which blocked JNK1/2 phosphorylation, could not alter the effect of COL2A1 (Supplementary Fig. 6a-c). *COL2A1* silencing confirmed that COL2A1 could regulate SMAD1^S206^ and ERK1/2 phosphorylation (Supplementary Fig. 6d).

Furthermore, COL2A1 was found to facilitate the SMAD1−ERK1/2 interaction (Fig. 6h, i). U0126 partially released COL2A1’s inhibition of SMAD1 nuclear import (Fig. 6j). Finally, COL2A1 was demonstrated to be capable of suppressing the binding between SMAD1 and SMAD4 (Fig. 6k, l).

### COL2A1 signaling was associated with OA cartilage degeneration and pathological changes of the subchondral bone

**Fig. 7:**
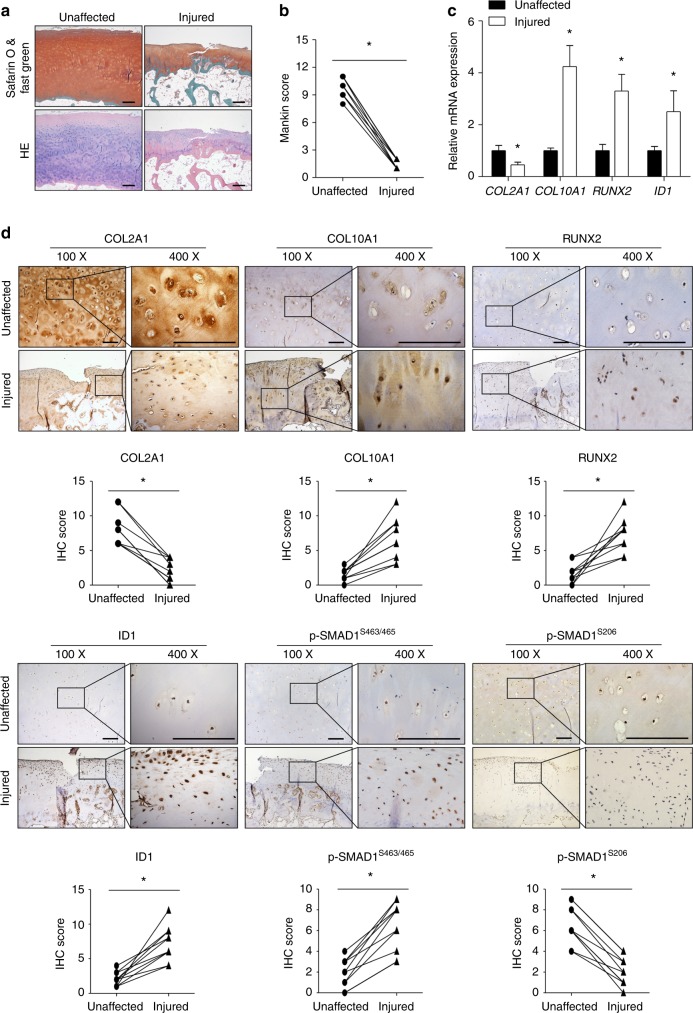
COL2A1 signaling was associated with OA cartilage degeneration and pathological changes of the subchondral bone. Both injured cartilage (mostly from the rim of the ulcer, comprising ~3–4 mm of the surrounding tissue) and paired macroscopically unaffected cartilage were collected from ten patients with knee OA. **a**, **b** Safranin O/Fast green staining, HE staining, and the Mankin score were evaluated. **c** The expression levels of *COL2A1*, *COL10A1*, *RUNX2*, and *ID1* were detected using qPCR in both injured cartilage and paired unaffected cartilage. **d** IHC staining of COL2A1, COL10A1, RUNX2, ID1, p-SMAD1^S463/465^, and p-SMAD1^S206^ was performed, and a semiquantitative analysis of IHC images was conducted. Data in (**c**) are presented as the mean ± SD (*n* = 10). *P < 0.05. OA, osteoarthritis; IHC, immunohistochemistry

Safranin O/Fast green staining, HE staining, and the Mankin score were evaluated in both injured cartilage (mostly from the rim of the ulcer, comprising ~3–4 mm of the surrounding tissue) and paired macroscopically unaffected cartilage distal to the damaged zone from ten patients with knee OA (Fig. 7a, b; Supplementary Table 3). The expression levels of COL2A1, p-SMAD1^S206^, p-ERK1/2, and p-ITGB1 were dramatically decreased in the injured cartilage, while the expression levels of COL10A1, RUNX2, ID1, and p-SMAD1^S463/465^ were significantly elevated (Fig. 7c, d; Supplementary Fig. 7a, b).

Moreover, we graded the pathological changes of the subchondral bone in 50 OA cartilage samples according to previous studies^40^ and analyzed their correlations with the protein levels of COL2A1, COL10A1, and p-SMAD1^S463/465^ (the protein levels of these molecules were detected, and the results are shown in Fig. 3k−m). We found that cartilage with more severe pathological changes in the subchondral bone displayed lower expression of COL2A1 and higher expression of COL10A1 and p-SMAD1^S463/465^ (Supplementary Fig. 7c-e), suggesting that the decrease in COL2A1 might also play a role in the subchondral bone damage of OA joints.

## Discussion

In our previous study, the *Col2a1* p.Gly1170Ser homozygous mutation was shown to cause chondrodysplasia by activating the ERS-UPR-apoptosis cascade.^21^ In addition to causing the ERS-UPR-apoptosis cascade, misfolded type II procollagen was restrained and degraded in the ER, which is in accordance with many other studies of the *COL2A1* (or *Col2a1*) mutation.^41–44^ Thus, Col2a1 secretion and assembly decreased sharply and led to a situation in which Col2a1 was nearly absent from the homozygous cartilage matrix.^21,41^ Furthermore, it was confirmed that Col2a1 loss, but not the ERS-UPR-apoptosis cascade, was the main reason for the enhanced chondrocyte hypertrophy and disturbed TGFβ/BMP pathway. The *Col2a1* p.Gly769Ser mutant mouse demonstrated significantly decreased Col2a1 protein and enhanced hypertrophic differentiation in chondrocytes, similar to what we found in our mouse model,^41^ which lends credence to our opinion that Col2a1 loss is responsible for chondrocyte hypertrophy. Because purified COL2A1, the soluble COL2A1 peptide, which has no structural function, can still inhibit chondrocyte hypertrophy in vitro, we suspected that the signaling activity of COL2A1 played a predominant role in regulating chondrocyte hypertrophy.

**Fig. 8:**
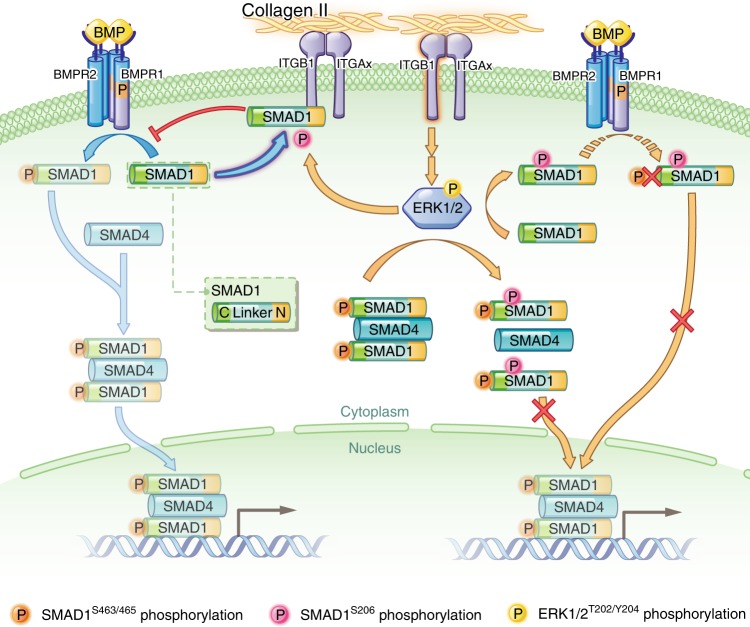
Functional mechanisms of COL2A1 on the BMP-SMAD1 pathway. The canonical BMP-SMAD1 pathway is initiated with the oligomerization of BMPR1 and BMPR2 induced by BMPs. BMPR2 phosphorylates BMPR1, and BMPR1, in turn, phosphorylates the C-terminus of SMAD1 (Ser463/465). C-terminal phosphorylated SMAD1 proteins partner with SMAD4 and translocate into the nucleus to initiate target gene transcription. ITGB1 is the major receptor for COL2A1, and upon its interaction with COL2A1, ITGB1 competes with BMPR1 for binding to SMAD1, thus inhibiting SMAD1^S463/465^ phosphorylation and nuclear import. COL2A1 can also regulate SMAD1 activity via ITGB1-induced ERK1/2 phosphorylation. Activated ERK1/2 phosphorylates the linker region (Ser206) of both unphosphorylated and C-terminal phosphorylated SMAD1. The linker region-phosphorylated SMAD1 is resistant to phosphorylation by BMPR1 at the C-terminal, while the interaction between the C-terminal-phosphorylated SMAD1 and SMAD4 can also be interrupted by a second phosphorylation of the linker region of SMAD1. As a result, SMAD1 activation can be repressed via various signaling pathways specifically in response to COL2A1. BMP, bone morphogenetic protein

This study illustrates the novel role of COL2A1 in regulating the chondrocyte transition between the quiescent state and hypertrophic differentiation. In healthy articular cartilage, chondrocytes remain in a postmitotic quiescent state, while in OA cartilage, some chondrocytes around OA cartilage lesions can be activated and undergo hypertrophy during the whole disease process.^4,5^ In the current study, we demonstrate that COL2A1 acts as an extracellular signaling molecule to inhibit chondrocyte hypertrophy. Upon interaction with COL2A1, ITGB1 receptors compete with BMP receptors to bind with SMAD1 and then inhibit SMAD1 activation and nuclear import. COL2A1 also activated ITGB1-induced ERK1/2 phosphorylation, and through ERK1/2−SMAD1 interaction, it further repressed SMAD1 activation, thus inhibiting BMP-SMAD1-mediated hypertrophic differentiation (Fig. 8). COL2A1 loss led to the release of BMP-SMAD1 signaling suppression, resulting in chondrocyte hypertrophy and the promotion of OA progression, while supplementation with COL2A1 reversed chondrocyte hypertrophy. COL2A1 is suggested to be crucial in the chondrocyte quiescent state-hypertrophic differentiation-transition: COL2A1 maintains the chondrocyte quiescent state, while loss of COL2A1 leads to hypertrophic differentiation. We revealed the detailed mechanisms underlying COL2A1’s regulation of the cartilaginous phenotype, in which ITGB1 and BMP-SMAD1 are the critical downstream effectors. Thus, our findings provide new insight into the regulation of cartilaginous phenotype and OA progression.

COL2A1 is the major component of the cartilage matrix, and its degradation is considered a typical pathological process in OA development.^3,9,45^ However, the current study suggests that COL2A1 degradation and reduction can initiate and promote OA progression by accelerating chondrocyte hypertrophy. We now introduce a disease-amplifying loop model to explain the effect of decreased COL2A1 on OA development. During the early stage of OA, once focal cartilage lesions develop, the proliferation and anabolism of surrounding chondrocytes are activated to repair the damage, during which BMP-SMAD1 is simultaneously activated and plays a critical role. However, the continuous activation of BMP-SMAD1 signaling also induces a portion of the surrounding chondrocytes to undergo hypertrophic differentiation, downregulating COL2A1 expression and upregulating MMPs that can break down the existing COL2A1. COL2A1 reduction prevents its suppression of the BMP-SMAD1 pathway and further accelerates chondrocyte hypertrophy, leading to the formation of the self-perpetuating disease-amplifying loop. Through this self-enhancing loop, chondrocyte hypertrophy and cartilage matrix destruction could propagate from focal damage to neighboring cartilage. In light of this disease model, we propose that the COL2A1 decrease plays a central role in OA development by acting as a main factor driving the aforementioned disease-amplifying loop. Targeting the key factors in this disease-amplifying loop may be a promising alternative OA therapy, considering that supplementation with functional COL2A1 directly into an OA joint remains difficult. The *COL2A1* p.G1170S mutation was demonstrated to be able to cause early-onset OA in a five-generation family,^46^ and several other *COL2A1* mutations have also been reported to be related to OA.^47–49^ Although *COL2A1* mutation is not frequently observed in human OA in the general population, considering the central role of cartilage matrix in OA development, studies of the effect of *COL2A1* mutation on OA could provide insight into the regulation of cartilage homeostasis and shed light on potential essential molecular mechanisms in OA. Mice with inactivation of one allele of the *Col2a1* gene showed softer articular cartilage, disturbed collagenous network, reduced subchondral bone volume, and increased susceptibility to OA, which adds credibility to the role of the COL2A1 decrease in OA development.^50^

Although ITGB1 signaling has been proven to suppress chondrocyte hypertrophy by both transgenic mouse models and in vitro studies, the detailed molecular mechanisms are unclear.^13,18^ The current study showed that the canonical BMP-SMAD1 pathway may mediate the effect of ITGB1 signaling on chondrocyte hypertrophy. The direct interaction between ITGB1 and SMAD1 was identified for the first time. Further study showed that COL2A1 facilitated ITGB1−SMAD1 interaction while simultaneously attenuating the BMPR1A/B−SMAD1 interaction. In this way, ITGB1 inhibited SMAD1^S463/465^ phosphorylation and nuclear translocation in response to COL2A1. We also confirmed that COL2A1 could downregulate SMAD1 activation via the ITGB1-ERK1/2 axis-induced SMAD1^S206^ phosphorylation. The current study not only reveals the mechanisms underlying ITGB1 regulation on chondrocyte hypertrophy but also partly explains how ITGB1 affects OA progression.

A new negative regulation mechanism on BMP-SMAD1 signaling was illustrated in this study: SMAD1 activity can be negatively regulated directly by the ITGB1 receptor in response to COL2A1. It has been well established that COL2A1 activates the TGFβ-SMAD2/3 pathway through a membrane signaling complex formed by type I TGFβ receptors and integrins.^10^ Although TGFβ-SMAD2/3 plays a role in inhibiting chondrocyte hypertrophy, TGFβ signaling alone seems insufficient for suppressing the onset of hypertrophy.^51,52^ Moreover, a shift in signaling dominance from TGFβ-SMAD2/3 to BMP-SMAD1/5/8 could induce AC hypertrophy.^53^ The current study also confirmed that the inhibitory effect of COL2A1 on chondrocyte hypertrophy was mainly mediated by the BMP-SMAD1 branch. In physiological conditions, upon interacting with ITGB1, the tuning modulation of COL2A1 acts to optimize the intensity and duration of both the BMP-SMAD1 signal and the TGFβ-SMAD2/3 signal and maintains the balance between them. Under pathological conditions, the tuning effect of COL2A1 is disturbed due to its decrease, BMP-SMAD1 signaling prevails, and chondrocytes undergo hypertrophy.

In conclusion, the current study demonstrated that COL2A1 acts as a potent suppressor for chondrocyte hypertrophy and OA progression through the negative regulation of SMAD1 activity.

## Materials and methods

### Animals

Detailed procedures for the construction of *Col2a1* p.Gly1170Ser knockin mice have been previously described.^21^ Animals were housed in a temperature- and humidity-controlled room under a 12-h light-and-dark cycle with food and water provided ad libitum.

### Source of human cartilage

Human cartilage samples were obtained from 60 OA patients classified as grades 3 and 4 according to the Kellgren and Lawrence osteoarthritis grading system. The patients underwent total knee arthroplasty at Sun Yat-sen Memorial Hospital of Sun Yat-sen University between 2014 and 2015. Cartilage samples were taken from ten patients (seven women and three men with a mean age of (68.10 ± 5.55) years) from both the injured area (mostly from the rim of the ulcer, comprising ~3–4 mm of the surrounding tissue) and the macroscopically unaffected area distal to the damaged zone. Cartilage samples were taken from another 50 patients (36 women and 14 men with a mean age of (67.72 ± 7.62) years) from only the injured area. Measurements of mRNA expression and histology were performed. The samples were kept frozen at −80 °C until use or placed in paraformaldehyde for the histological study.

### Antibodies and reagents

The following antibodies (Abs) were purchased from Cell Signaling Technology (CST, Danvers, MA, USA): p-SMAD1 (Ser206), p-SMAD2 (Ser465/467), p-SMAD3 (Ser423/425), SMAD2/3, HA-Tag, p-ERK1/2 (Thr202/Tyr204), ERK1/2, p-MAPK14 (Thr180/Tyr182), MAPK14, and p-JNK1/2 (Thr183/Tyr185). Abs against GAPDH, RUNX2, COL10A1, MMP13, SMAD1, SMAD4, ITGB1, JNK1/2, THOC1, p-SMAD1 (Ser463/Ser465), and anti-phospho-serine/threonine antibody were from Abcam (Cambridge, UK). Abs against COL2A1, BMPR1A, and BMPR1B were obtained from Santa Cruz Biotechnology (Dallas, TX, USA). p-ITGB1 (Thr788) and Flag-Tag Abs were obtained from Sigma-Aldrich (St. Louis, MO, USA). Goat anti-rabbit IgG H&L (HRP) and goat anti-mouse IgG H&L (HRP) secondary antibodies were purchased from Thermo Scientific (Waltham, MA, USA). Goat anti-rabbit IgG (H+L), F(ab′)2 fragment (Alexa Fluor® 555 Conjugate) secondary antibodies were from CST. Anti-human IgG antibody was purchased from Abcam.

Blocking antibodies against human integrin beta1, recombinant human interleukin-1 beta, and recombinant human BMP2 were obtained from R&D systems. Purified COL2A1 was purchased from Chondrex, Inc. (Redmond, WA, USA). U0126 and SP600125 were from CST. SB505124 was obtained from MedChemExpress (Monmouth Junction, NJ, USA).

### Cell culture

The human chondrosarcoma cell lines SW1353 and Hs819.T were purchased from American Type Culture Collection (Manassas, VA, USA) and the mouse chondrogenic cell line ATDC5 was purchased from RIKEN Cell Bank (Tsukuba, Japan). The 293T cell line was purchased from the China Center for Type Culture Collection (Wuhan, China). SW1353 and ATDC5 were cultured in Dulbecco’s modified Eagle medium (DMEM)/F-12 medium (HyClone, Logan City, UT, USA) supplemented with 10% fetal bovine serum (FBS) (HyClone). Hs819.T and 293 T cells were cultured in DMEM medium (HyClone) supplemented with 10% FBS.

Mouse primary chondrocytes were isolated from the articular cartilage of 12-week-old C57BL/6 mice. Human ACs were obtained from ScienCell (Carlsbad, CA, USA). Both mouse and human chondrocytes were cultured in DMEM/F-12 supplemented with 10% FBS.

### Isolation and culture of human bone marrow-derived MSCs

MSCs were isolated and purified from bone marrow obtained from healthy volunteer donors using density-gradient centrifugation, as described previously.^54^ Cells were resuspended in low-glucose DMEM (Gibco, Waltham, MA, USA) with 10% FBS, seeded and incubated at 37 °C/5% CO_2_. After 48 h, nonadherent cells were removed by replacing the medium. Then, the medium was replaced every 3 d. When the cells reached (80–90)% confluence, they were trypsinized, counted, and reseeded as the first passage. Cells from passages 3–6 were used for subsequent experiments.

### Chondrogenic and hypertrophic differentiation of human MSCs

A high-density pellet culture system was applied for the chondrogenic differentiation of human MSCs, as described previously.^54^ Cells were induced for chondrogenic predifferentiation for 14 d in pellet culture (200 000 cells/pellet) in chondrogenic medium consisting of high-glucose DMEM with 50 μg·mL^−1^ ascorbate acid 2-phosphate (Sigma-Aldrich), 40 μg·mL^−1^ proline (Sigma-Aldrich), 100 nmol·L^−1^ dexamethasone (Sigma-Aldrich), 10 ng·mL^−1^ recombinant human TGFβ3 (R&D systems), and 1% ITS Universal Culture Supplement Premix (BD Biosciences, San Jose, CA, USA).

To further induce hypertrophic differentiation, chondrogenic differentiated pellets were exposed to hypertrophic differentiation medium consisting of high-glucose DMEM with 50 μg·mL^−1^ ascorbate acid 2-phosphate, 40 μg·mL^−1^ proline, 1 nmol·L^−1^ dexamethasone, 1% ITS Universal Culture Supplement Premix, and 1 nmol·L^−1^ triiodothyronine (Sigma-Aldrich) for 14 d as described previously.^52^ The medium was changed every 3 d.

### Real-time RT-PCR assay

Real-time PCR was performed on a Roche LightCycler 480 System (Roche, Basel, Switzerland) using SYBR Green Real-time PCR Master Mix (TOYOBO, Osaka, Japan). The primer sequences used in this study are listed in Supplementary Table 4. Each reaction was processed in triplicate, and an average ΔCt value from the whole group was taken. The relative expression levels of each gene were obtained using the 2^−ΔΔCt^ method.

### Real-time PCR array

Total RNA from cultured mouse primary chondrocytes was extracted using an RNeasy plus micro kit (Qiagen, Duesseldorf, Germany). The high-quality RNA from 12 samples (3 genotypes × 4 different litters) was converted into cDNA with an RT2 First Strand cDNA Kit (SABiosciences, Duesseldorf, Germany). Osteogenesis-related gene expression was determined by using the PCR Array of PAMM-026 (SABiosciences; the list of genes is available on its website) and the 7500 Real-Time PCR system (Applied Biosystems, Waltham, USA) according to the manufacturer’s instructions. Statistical analysis was performed using paired-samples tests, and the fold changes were calculated using the 2^−ΔΔCt^ method with the Web-Based PCR Array Data Analysis system (SABiosciences). Differentially expressed genes were identified by a fold change of >2 and a change in *P* value < 0.05 for increased expression or a fold change of <0.5 and a change in *P* value < 0.05 for decreased expression. The Database for Annotation, Visualization, and Integrated Discovery (DAVID), Bioinformatics Resources 6.7 (https://david.ncifcrf.gov/home.jsp) was also used for data mining.

### Concentration and quality score measurement of RNA samples

Total RNA was isolated from cells using RNAiso Plus reagent (TaKaRa, Kusatsu, Japan) and detected by RNA LabChip (PerkinElmer, Inc., Waltham, MA, USA) and an RNA Reagent kit (PerkinElmer, Inc.) on a LabChip GX Touch HT Bioanalyzer (PerkinElmer, Inc.) according to the manufacturer’s protocols. The concentration and RNA quality score (RQS) of all RNA samples are listed in Supplementary Table 5.

### Plasmids, siRNAs, and transfection

To silence *Col2a1* gene expression in the ATDC5 cell line, mouse shRNA sequences were cloned into a lentiviral transfer plasmid, hU6-MCS-CMV-Puromycin (GV112), to generate hU6-MCS-CMV-Puromycin-Col2a1-shRNA1, hU6-MCS-CMV-Puromycin-Col2a1-shRNA2, and they were produced by Shanghai GeneChem Co., Ltd. (Shanghai, China). Control scramble shRNA (LVCON054) was also constructed. Lentiviral infection was performed according to the operations manual. The pCMV3-FLAG-negative control vector plasmid (CV012), pCMV3-FLAG-SMAD1 plasmid (HG10715-CF), and pCMV3-HA-ITGB1 plasmid (HG10587-CF) were obtained from Sino Biological Inc. The pCMV3-Flag-CA-BMPR1A (constitutively activated BMPR1A) plasmid and pCMV3-Flag-CA-TGFβR1 plasmid were constructed according to previous studies.^55,56^ All siRNAs were provided by Ribobio (Guangzhou, China). Cells at 30% confluence were employed for plasmid or siRNA transfection assays using Lipofectamine 3000 reagent (Invitrogen).

### Preparation of nuclear and cytoplasmic extracts

Nuclear and cytoplasmic extracts were prepared using nuclear and cytoplasmic extraction reagents (Thermo Scientific), following the manufacturer’s recommended protocol.

### Immunoblotting analysis

Cells were washed twice with ice-cold phosphate buffer saline, harvested, and resuspended in RIPA Lysis Buffer (Beyotime, Shanghai, China) plus protease inhibitor cocktail (MedChemExpress). Cell lysates were obtained by centrifugation at 12 000 r·min^−1^ for 10 min at 4 °C. Equal amounts of each sample were subjected to SDS-polyacrylamide gel electrophoresis (PAGE) and transferred to polyvinylidene fluoride membranes (Millipore, Boston, MA, USA). Membranes were blocked with 5% nonfat dry milk for 1 h at room temperature and then incubated with the designated antibodies. Antibody-specific labeling was revealed by incubation with HRP-conjugated secondary antibodies for 1 h and visualized with an electrochemiluminescence (ECL) kit (Millipore). Images were captured and analyzed with an ImageQuant Las 4000mini imaging system (GE Healthcare Life Science, Chicago, IL, USA). All immunoblotting assays were conducted with three biological replicates, and representative images are shown.

### Purification of SMAD1 protein from 293T cells

Flag-tagged SMAD1 was expressed in a mammalian cell expression system. Lysates were prepared from 5 × 10^7^ 293T cells transfected with pCMV3-C-FLAG-SMAD1 plasmid using RIPA Lysis Buffer (Beyotime). Lysates were incubated with 20 μL Anti-Flag affinity agarose (Sigma-Aldrich) overnight at 4 °C. Beads containing affinity-bound proteins were washed seven times with 5 mL wash buffer (300 mmol·L^−1^ NaCl, 20 mmol·L^−1^ Hydroxyethyl piperazine ethylsulfonic acid (HEPES), 1 mmol·L^−1^ Ethylene diamine tetraacetic acid (EDTA), 1 mmol·L^−1^ Ethylene glycol tetraacetic acid (EGTA), 2% glycerol, pH 7.4, and 0.1% NP-40), followed by elution with 100 μL of Poly Flag peptides (0.4 μg·μL^−1^, Biotool, Houston, TX, USA). The eluate was collected and examined by SDS-PAGE, Coomassie blue staining, and mass spectrometry analysis.

### Immunoprecipitation

During endogenous immunoprecipitation, cells were lysed with lysis buffer containing 150 mmol·L^−1^ NaCl, 20 mmol·L^−1^ HEPES, 1 mmol·L^−1^ EDTA, 1 mmol·L^−1^ EGTA, 10% glycerol, pH 7.4, and 0.1% NP-40. The same amount of protein from each sample was incubated with specific antibodies and protein G agarose (Millipore) overnight at 4 °C. The agarose-bound immunoprecipitates were washed seven times with immunoprecipitation wash buffer (150 mmol·L^−1^ NaCl, 20 mmol·L^−1^ HEPES, 1 mmol·L^−1^ EDTA, 1 mmol·L^−1^ EGTA, 2% glycerol, pH 7.4, and 0.1% NP-40) and collected by centrifugation. Samples were subjected to SDS-PAGE and immunoblotting analysis after the addition of 30 μL of sample buffer (62 mmol·L^−1^ Tris-HCl, 1.25% (w/v) SDS, 10% (v/v) glycerol, 3.75% (v/v) mercaptoethanol, and 0.05% (w/v) bromophenol blue, pH 6.7) and denaturation.

Using lysis buffer, lysates were prepared from cells transfected with plasmids during exogenous immunoprecipitation. The same amount of protein from each sample was incubated with 20 μL anti-Flag affinity agarose or anti-HA affinity agarose (Biotool) overnight at 4 °C. The agarose-bound immunoprecipitates were washed seven times with immunoprecipitation wash buffer and collected by centrifugation. Samples were then subjected to immunoblotting analysis after the addition of 30 μL of sample buffer and denaturation.

### IHC analysis

Paraffin sections (4 μm) were prepared, and IHC was performed with a Histostain-Plus kit (ZSGB-BIO, Beijing, China). The primary antibodies included anti-COL2A1, COL10A1, RUNX2, ID1, and p-SMAD1^S463/465^ antibodies. A DAB Horseradish Peroxidase Color Development Kit (ZSGB-BIO) was used for detection. Immunostaining evaluations were performed independently by experimenters blinded to sample identity. The staining intensity was scored as follows: 0 (negative), 1 (weakly positive), 2 (moderately positive), and 3 (strongly positive). The percent of positivity was also scored according to 5 categories: 0 (<5%), 1 (5%−25%), 2 (25%−50%), 3 (50%−75%), and 4 (>75%). Then, the value of the percent positivity score was multiplied by the staining intensity score to generate final expression scores, which ranged from 0 to 12.

### TUNEL labeling assay

A TUNEL assay was performed according to the manufacturer’s instructions (MBL, Nagoya, Japan), and photographs were captured using a Leica DMI4000B microscope (Leica, Wetzlar, Germany). The percentages of TUNEL-positive cells relative to 4',6-diamidino-2-phenylindole (DAPI)-stained cells were calculated. Three independent experiments were conducted and calculated for each experimental group.

### Immunofluorescence and confocal microscopy analysis

Cells were treated with purified collagen type II for 24 h, fixed with 4% paraformaldehyde, pretreated with 1% Triton X-100 and 0.5% bovine serum albumin (BSA) in phosphate buffer saline tween-20 (PBST), and blocked with 10% BSA in PBST for 30 min at room temperature. Cells were labeled with anti-SMAD1 antibody and treated with a secondary antibody, Alexa Fluor 555-conjugated goat anti-rabbit IgG, and then stained with DAPI. Signals were captured with a Zeiss LSM780 confocal laser scanning microscope (Zeiss, Jena, Germany).

### Statistical analysis

The results are given as the mean ± standard deviation (SD). Statistical analysis was performed using the two-tailed independent Student’s *t* test for comparisons of two independent groups, two-tailed paired-sample *t* test for comparisons of two matched groups, and one-way ANOVA followed by Dunnett’s post hoc test for multiple comparisons. In all cases, a *P* value less than 0.05 was considered statistically significant. All statistical analyses were conducted with the SPSS 13.0 statistical software package.

### Study approval

All experimental procedures involving animals met the relevant guidelines for the humane care of laboratory animals and were approved by the Institutional Animal Care and Use Committee of Sun Yat-sen University. For human studies, prior patients’ consent and approval from the Institutional Research Ethics Committee of Sun Yat-sen University were obtained.

## Supplementary information

Supplementary data
