# Supplementary material for: Collagen type II suppresses articular chondrocyte hypertrophy and osteoarthritis progression by promoting integrin β1−SMAD1 interaction
Source: Bone Res. 2019 Mar 6;7:8. doi: 10.1038/s41413-019-0046-y (PMC6403405; doi:10.1038/s41413-019-0046-y)
Supplement: Supplementary file 1 — Supplementary data [file 41413_2019_46_MOESM1_ESM.docx]

**Collagen type II suppresses articular chondrocyte hypertrophy and osteoarthritis progression by promoting integrin β1-SMAD1 interaction**

**Supplementary Figures and figure legends**

**Supplementary Fig. 1**

**
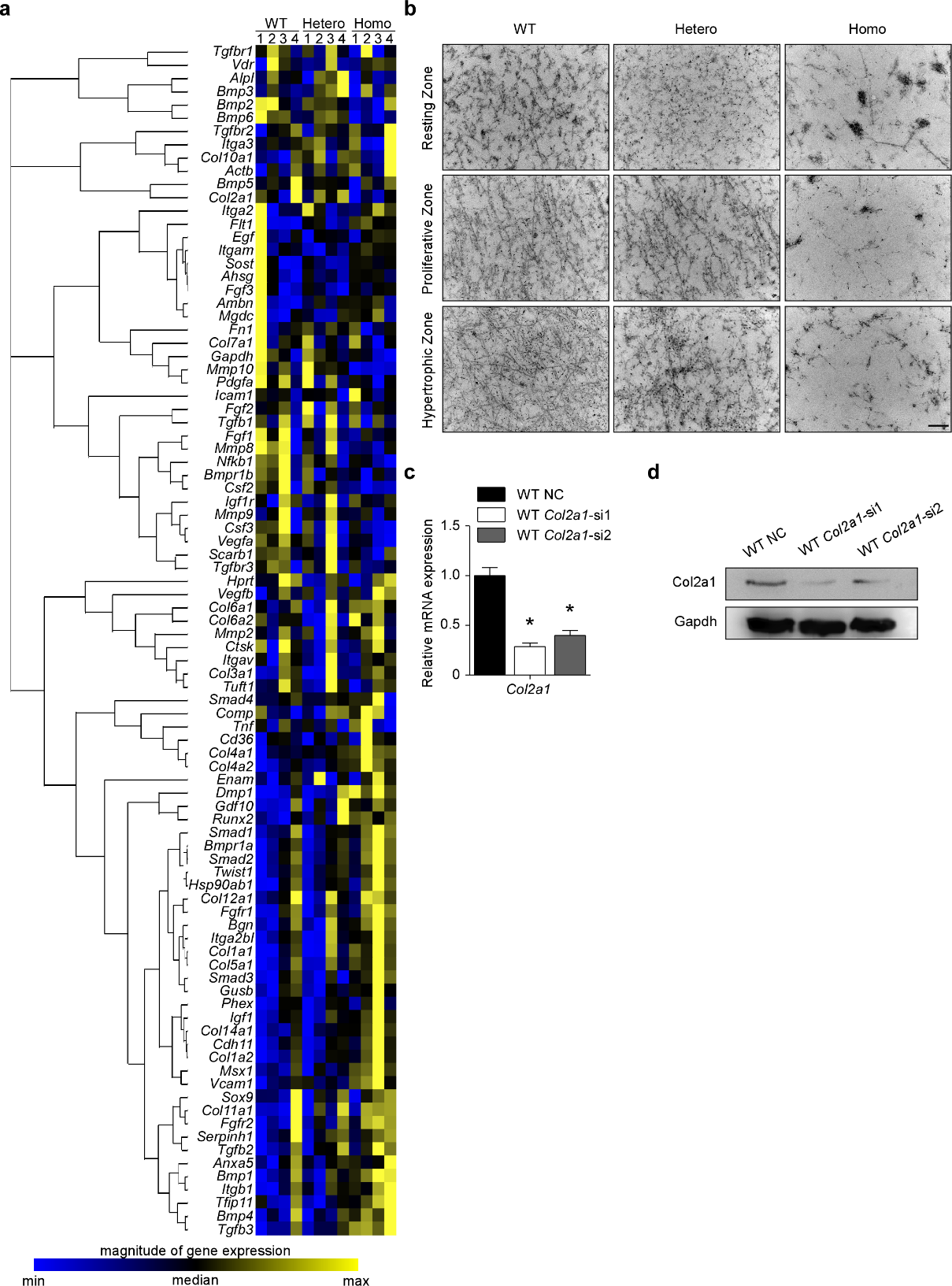
**

**Supplementary Fig.** **1** Chondrocytes from *Col2a1* mutant mice underwent enhanced hypertrophic differentiation due to the Col2a1 loss. **a** The *Col2a1* p.Gly1170Ser knock-in mice were constructed and the primary chondrocytes were isolated from the articular cartilage of embryos of all three genotypes and cultured for 7 d. The qPCR array analysis was performed. The cluster graph and heat map were constructed using a Web-Based PCR Array Data Analysis system (SABiosciences). **b** Transmission electron microscope analysis of the cartilage matrix of the growth plates from *Col2a1* p.Gly1170Ser mutant mice. Scale bars: 200 nm. **c, d** After silencing of *Col2a1* by siRNAs in wild type chondrocytes, the mRNA (**c**) and protein (**d**) levels of Col2a1 were detected. Data in **c** are presented as mean ± SD (n = 3). **P* < 0.05.

**Supplementary Fig. 2**


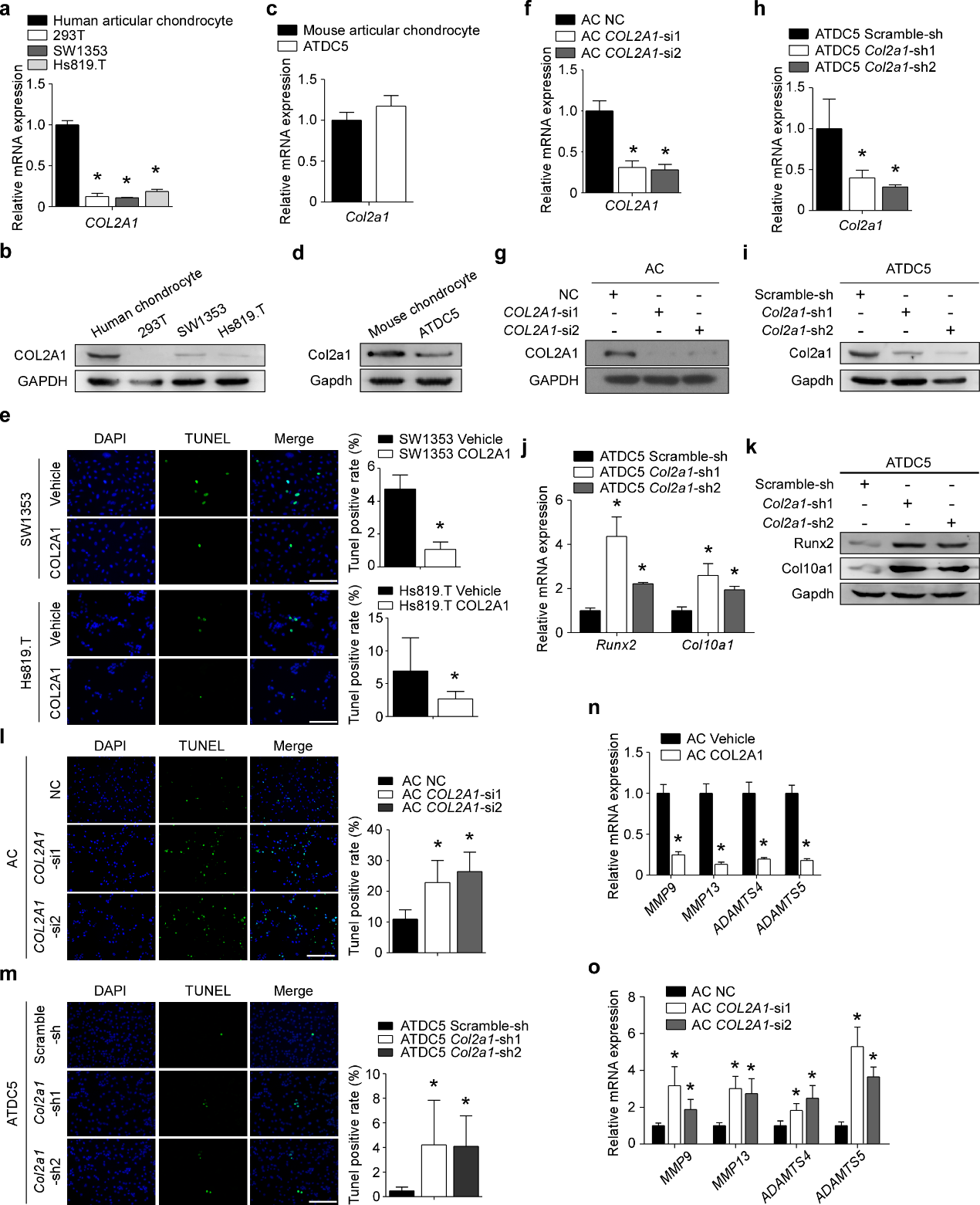


**Supplementary Fig.** **2** COL2A1 exerted suppression on chondrocyte hypertrophy and production of matrix degrading enzymes. **a, b** Expression of COL2A1 by qPCR (**a**) and immunoblotting (**b**) in 293T, SW1353, and Hs819.T cells compared with human articular chondrocyte. **c, d** Expression of Col2a1 by qPCR (**c**) and immunoblotting (**d**) in ATDC5 cells compared with mouse articular chondrocyte. **e** SW1353 and Hs819.T cells were treated with 10 ng/mL IL-1β for 24 h to induce apoptosis, and 100 μg/mL COL2A1 or vehicle (0.05M acetic acid) was added simultaneously. TUNEL assay was conducted, and positive rates were statistically analyzed (right panel, 3 independent experiments were conducted for each group). Scale bars: 200 μm. **f, g** Human articular chondrocytes were transfected with *COL2A1* siRNAs or negative control siRNA, and mRNA (**f**) and protein (**g**) levels of COL2A1 were detected. **h-k** ATDC5 were transfected with *Col2a1*-shRNAs or Scramble-shRNA, and mRNA (**h**) and protein (**i**) levels of Col2a1 were detected. And expressions of Runx2 and Col10a1 were detected by qPCR (**j**) and immunoblotting (**k**). **l** Human articular chondrocytes transfected with *COL2A1* siRNAs or negative control siRNA were treated with IL-1β. TUNEL assay was conducted and positive rates were statistically analyzed (right panel, 3 independent experiments were conducted for each group). Scale bars: 200 μm. **m** ATDC5 cells transfected with *Col2a1*-shRNAs or Scramble-shRNA were treated with IL-1β. TUNEL assay was conducted and positive rates were statistically analyzed (right panel, 3 independent experiments were conducted for each group). Scale bars: 200 μm. **n** Human articular chondrocytes were treated with vehicle (0.05 M acetic acid) or 100 μg/mL COL2A1 for 48 h, the expression of *MMP9*, *MMP13*, *ADAMTS4*, and *ADAMTS5* was detected by qPCR. **o** *COL2A1* was silenced in human articular chondrocytes, the expression of *MMP9*, *MMP13*, *ADAMTS4*, and *ADAMTS5* was detected by qPCR. Data in **a**, **c**, **e**, **f**, **h**, **j**, **l**, **m**, **n** and **o** are presented as mean ± SD (n = 3). **P* < 0.05. AC, articular chondrocytes.

**Supplementary Fig. 3**


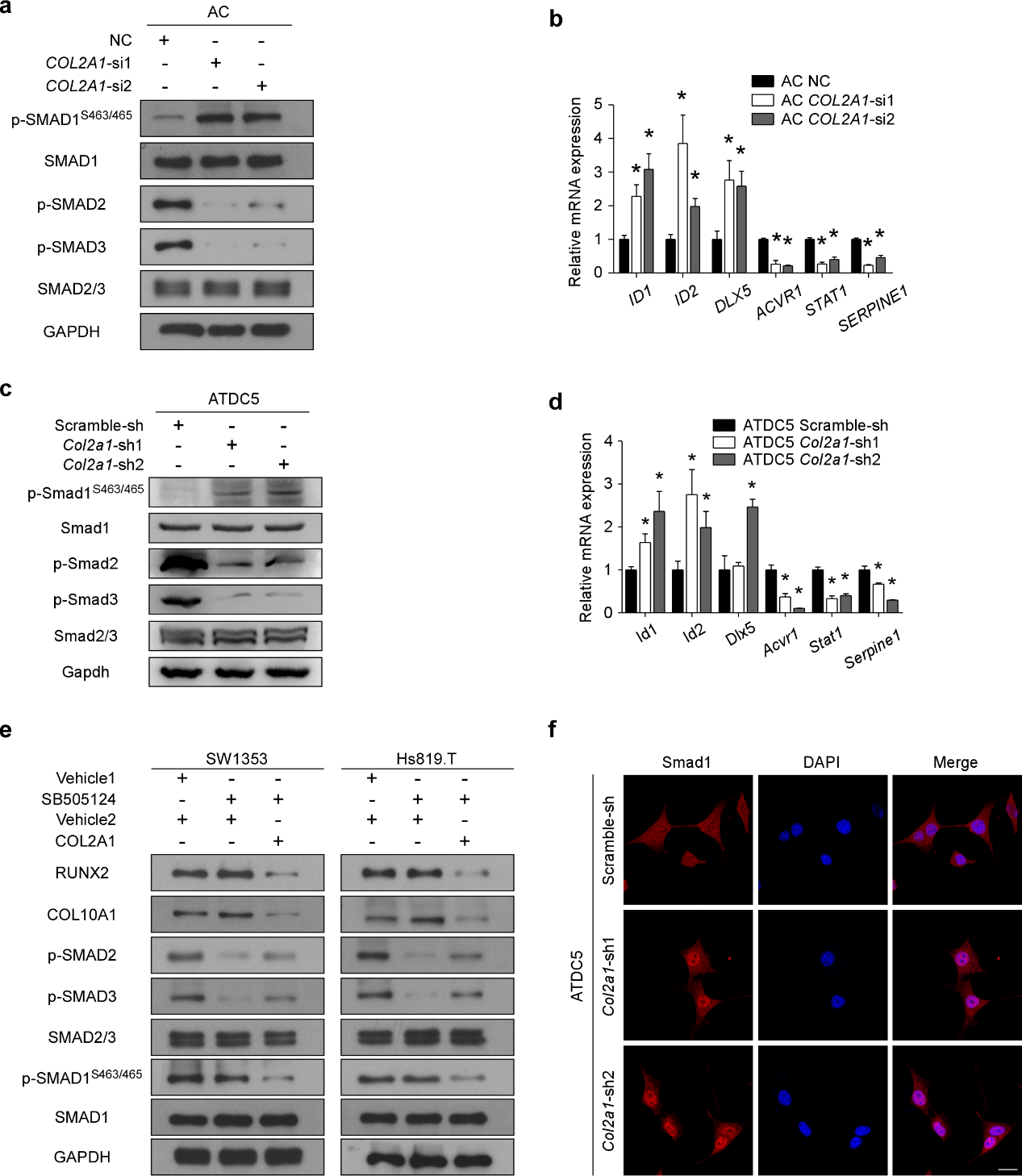


**Supplementary Fig. 3** COL2A1 suppressed chondrocyte hypertrophy through the regulation of the BMP-SMAD1 pathway. **a, b** *COL2A1* was silenced in human articular chondrocyte. Expressions of p-SMAD1^S463/465^, p-SMAD2, p-SMAD3, SMAD1 and SMAD2/3 were detected by immunoblotting (**a**), and expressions of *ID1*, *ID2*, *DLX5*, *ACVR1*, *STAT1*, and *SERPINE1* were detected by qPCR (**b**). **c, d** *Col2a1* was silenced in ATDC5 cells. Expressions of p-Smad1^S463/465^, p-Smad2, p-Smad3, Smad1 and Smad2/3 were detected by immunoblotting (**c**), and expressions of *Id1*, *Id2*, *Dlx5*, *Acvr1*, *Stat1*, and *Serpine1* were detected by qPCR (**d**). **e** SW1353 and Hs819.T cells were pretreated with 5 μM SB505124 or vehicle 1 (DMSO) for 0.5 h and then treated with 100 μg/mL COL2A1 or vehicle 2 (0.05M acetic acid) for 1 h. Expressions of RUNX2, COL10A1, p-SMAD2, p-SMAD3, SMAD2/3, p-SMAD1^S463/465^, and SMAD1 were detected by immunoblotting. **f** Confocal laser scanning was performed to demonstrate Smad1 nuclear localization in ATDC5 cells transfected with *Col2a1*-shRNAs or Scramble-shRNA. Scale bars: 20 μm. Data in **b** and **d** are presented as mean ± SD (n = 3). **P* < 0.05. AC, articular chondrocytes.

**Supplementary Fig. 4**


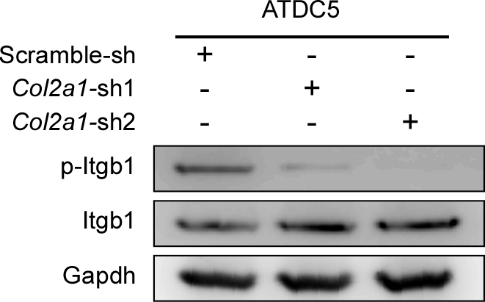


**Supplementary Fig.** **4** ITGB1 receptor mediated the effect of COL2A1 on BMP pathway and chondrocyte hypertrophy. *Col2a1* was silenced in ATDC5 cells, and expressions of p-Itgb1 and Itgb1 were detected by immunoblotting.

**Supplementary Fig. 5**


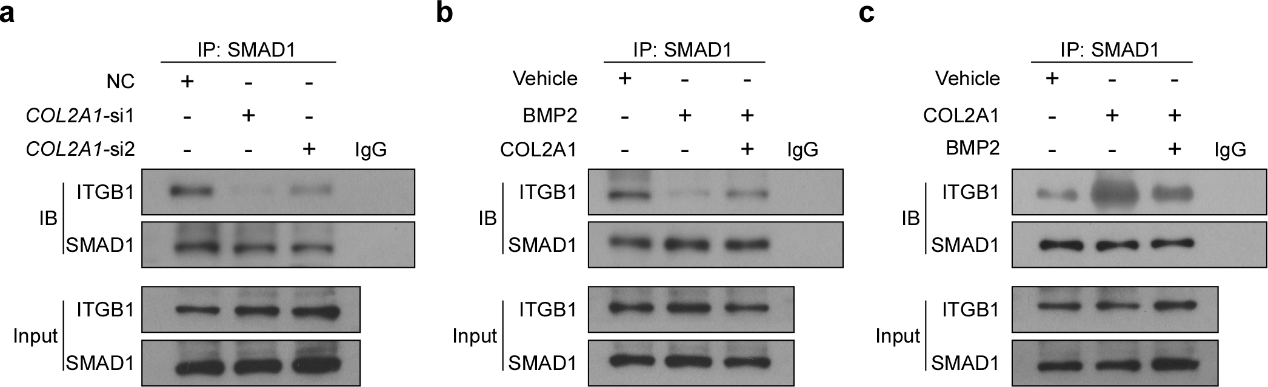


**Supplementary Fig. 5** COL2A1 repressed BMP-SMAD1 signaling activation through facilitating ITGB1-SMAD1 interaction and weakening BMPR1A/B-SMAD1 interaction. **a** Immunoprecipitation with the anti-SMAD1 antibody was performed in extracts of human articular chondrocytes with *COL2A1* silenced or not, and then immunoblotting was conducted with anti-ITGB1 and anti-SMAD1 antibodies. **b, c** Human articular chondrocytes were treated with vehicle (0.05M acetic acid), 10 ng/mL BMP2, or 100 μg/mL COL2A1, and immunoprecipitation was carried out with anti-SMAD1 antibody and followed by immunoblotting with anti-ITGB1 antibody and anti-SMAD1 antibody.

**Supplementary Fig. 6**


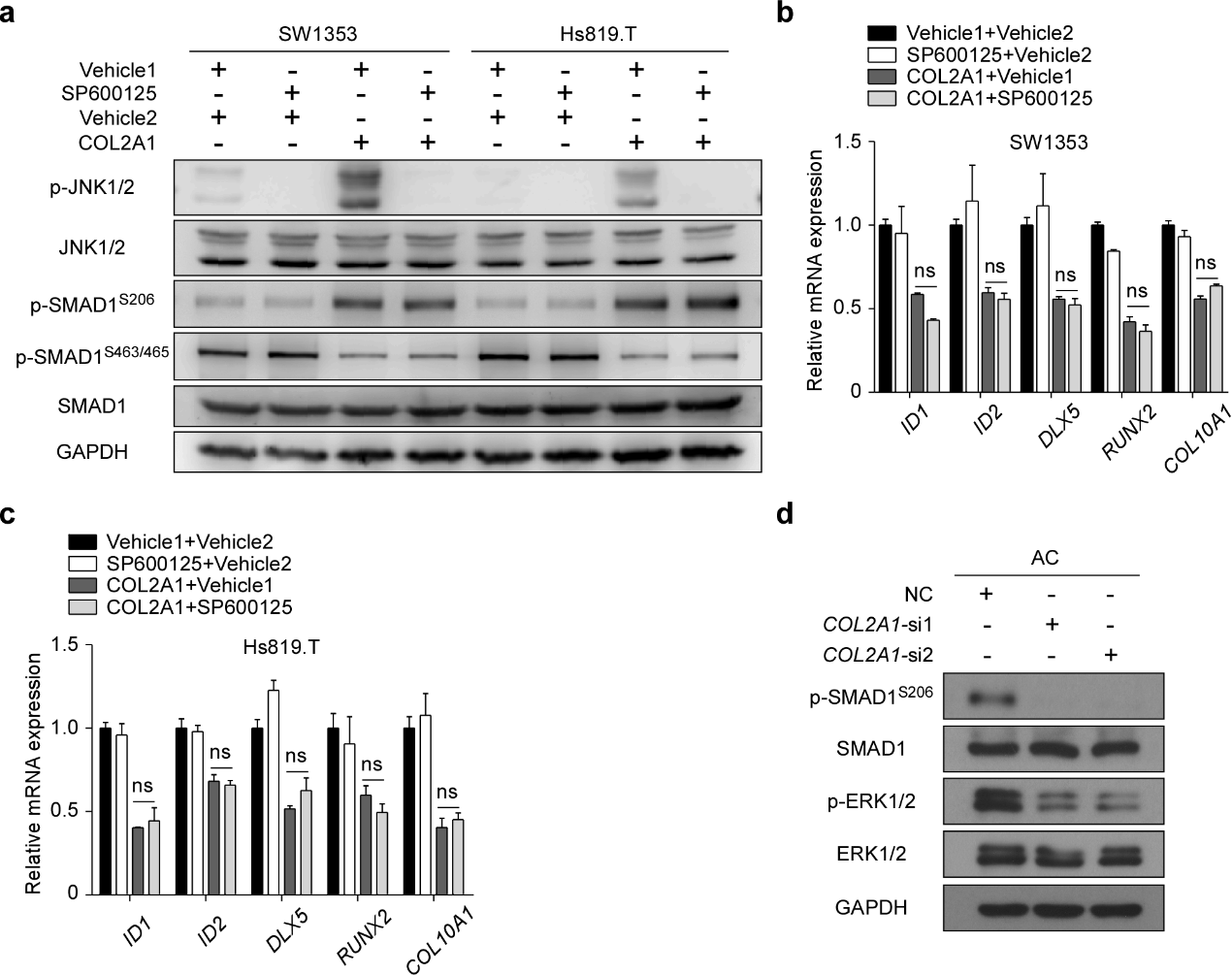


**Supplementary Fig.** **6** COL2A1 phosphorylated SMAD1^S206^ by activating ERK1/2, which exerted a negative influence on BMP-SMAD1 activity. **a-c** Protein levels of p-JNK1/2, JNK1/2, p-SMAD1^S206^, p-SMAD1^S463/465^ and SMAD1 were detected by immunoblotting (**a**), and mRNA levels of *Id1*, *Id2*, *Dlx5*, *Runx2*, and *Col10a1* were detected by qPCR in SW1353 (**b**) and Hs819.T cells (**c**) pretreated with 10 μM SP600125 or vehicle 1 (DMSO) for 1 h and then treated with 100 μg/mL COL2A1 or vehicle 2 (0.05M acetic acid) for 1 h. **d** *COL2A1* was silenced in human articular chondrocyte, and the expressions of p-SMAD1^S206^, SMAD1, p-ERK1/2, and ERK1/2 were detected by immunoblotting. Data in **b** and **c** are presented as mean ± SD (n = 3). ns, no significance. AC, articular chondrocytes.

**Supplementary Fig. 7**


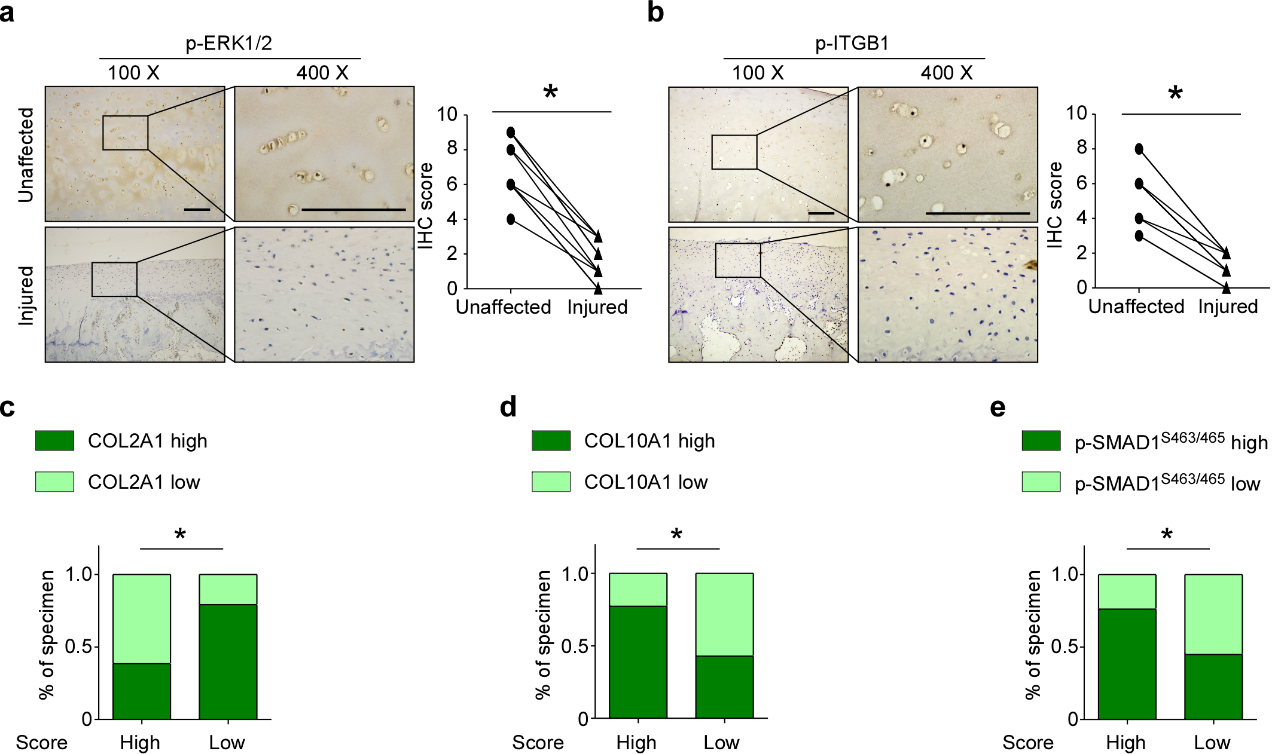


**Supplementary Fig.** **7** COL2A1 signaling was associated with OA cartilage degeneration and pathological changes of the subchondral bone. **a, b** Both the injured cartilage and the paired macroscopically unaffected cartilage from 10 patients with knee OA were collected. IHC staining of p-ERK1/2 (**a**) and p-ITGB1 (**b**) were performed, and semi quantitative analysis of IHC images was conducted. **c-e** The pathological changes of the subchondral bone in 50 OA cartilage samples were graded, the correlations between the score and the protein expressions of COL2A1 (**c**), COL10A1 (**d**), and p-SMAD1^S463/465^ (**e**) were examined. **P* < 0.05.

**Supplementary tables**

| **Supplementary Table 1. Relative gene expression in heterozygotes and homozygotes compared with wild types** | | | | | |
| --- | --- | --- | --- | --- | --- |
| Gene | GeneBank ID | Hetero | | Homo | |
|  |  | *P* Value | Fold Change | *P* Value | Fold  Change |
| *Ahsg*  *Alpl*  *Ambn*  *Anxa5*  *Bgn*  *Bmp1*  *Bmp2*  *Bmp3*  *Bmp4*  *Bmp5*  *Bmp6*  *Bmpr1a*  *Bmpr1b*  *Cd36*  *Cdh11*  *Col10a1*  *Col11a1*  *Col12a1*  *Col14a1*  *Col1a1*  *Col1a2*  *Col2a1*  *Col3a1*  *Col4a1*  *Col4a2*  *Col5a1*  *Col6a1*  *Col6a2*  *Col7a1*  *Comp*  *Csf2*  *Csf3*  *Ctsk*  *Dmp1*  *Egf*  *Enam*  *Fgf1*  *Fgf2*  *Fgf3*  *Fgfr1*  *Fgfr2*  *Flt1*  *Fn1*  *Gdf10*  *Icam1*  *Igf1*  *Igf1r*  *Itga2*  *Itga2b*  *Itga3*  *Itgam*  *Itgav*  *Itgb1*  *Mmp10*  *Mmp2*  *Mmp8*  *Mmp9*  *Msx1*  *Nfkb1*  *Pdgfa*  *Phex*  *Runx2*  *Scarb1*  *Serpinh1*  *Smad1*  *Smad2*  *Smad3*  *Smad4*  *Sost*  *Sox9*  *Tfip11*  *Tgfb1*  *Tgfb2*  *Tgfb3*  *Tgfbr1*  *Tgfbr2*  *Tgfbr3*  *Tnf*  *Tuft1*  *Twist1*  *Vcam1*  *Vdr*  *Vegfa*  *Vegfb*  *Gusb*  *Hprt*  *Hsp90ab1*  *Actb* | NM_013465  NM_007431  NM_009664  NM_009673  NM_007542  NM_009755  NM_007553  NM_173404  NM_007554  NM_007555  NM_007556  NM_009758  NM_007560  NM_007643  NM_009866  NM_009925  NM_007729  NM_007730  NM_181277  NM_007742  NM_007743  NM_031163  NM_009930  NM_009931  NM_009932  NM_015734  NM_009933  NM_146007  NM_007738  NM_016685  NM_009969  NM_009971  NM_007802  NM_016779  NM_010113  NM_017468  NM_010197  NM_008006  NM_008007  NM_010206  NM_010207  NM_010228  NM_010233  NM_145741  NM_010493  NM_010512  NM_010513  NM_008396  NM_010575  NM_013565  NM_008401  NM_008402  NM_010578  NM_019471  NM_008610  NM_008611  NM_013599  NM_010835  NM_008689  NM_008808  NM_011077  NM_009820  NM_016741  NM_009825  NM_008539  NM_010754  NM_016769  NM_008540  NM_024449  NM_011448  NM_018783  NM_011577  NM_009367  NM_009368  NM_009370  NM_009371  NM_011578  NM_013693  NM_011656  NM_011658  NM_011693  NM_009504  NM_009505  NM_011697  NM_010368  NM_013556  NM_008302  NM_007393 | \| 0.50 \| \| --- \| \| 0.77 \| \| 0.64 \| \| 0.78 \| \| 0.89 \| \| 0.88 \| \| 0.73 \| \| 0.05 \| \| 0.64 \| \| 0.58 \| \| 0.32 \| \| 0.79 \| \| 0.25 \| \| 0.49 \| \| 0.70 \| \| 0.30 \| \| 0.60 \| \| 0.46 \| \| 0.82 \| \| 0.93 \| \| 0.86 \| \| 0.81 \| \| 0.47 \| \| 0.54 \| \| 0.81 \| \| 0.96 \| \| 0.69 \| \| 0.43 \| \| 0.42 \| \| 0.75 \| \| 0.83 \| \| 0.54 \| \| 0.32 \| \| 0.02 \| \| 0.35 \| \| 0.92 \| \| 0.44 \| \| 0.98 \| \| 0.31 \| \| 0.78 \| \| 0.78 \| \| 0.65 \| \| 0.83 \| \| 0.52 \| \| 0.50 \| \| 0.76 \| \| 0.61 \| \| 0.90 \| \| 0.79 \| \| 0.97 \| \| 0.20 \| \| 0.63 \| \| 0.92 \| \| 0.64 \| \| 0.82 \| \| 0.71 \| \| 0.32 \| \| 0.63 \| \| 0.37 \| \| 0.31 \| \| 0.81 \| \| 0.43 \| \| 0.46 \| \| 0.93 \| \| 0.57 \| \| 0.71 \| \| 0.63 \| \| 0.81 \| \| 0.85 \| \| 0.73 \| \| 0.50 \| \| 0.87 \| \| 0.49 \| \| 0.82 \| \| 0.65 \| \| 0.82 \| \| 0.96 \| \| 0.63 \| \| 0.47 \| \| 0.95 \| \| 0.81 \| \| 0.65 \| \| 0.30 \| \| 0.89 \| \| 0.60 \| \| 0.22 \| \| 0.75 \| \| 0.90 \| | \| 0.95 \| \| --- \| \| 1.40 \| \| 3.46 \| \| 1.01 \| \| 1.11 \| \| 1.12 \| \| 0.98 \| \| 1.59 \| \| 0.98 \| \| 0.93 \| \| 0.87 \| \| 1.01 \| \| 0.81 \| \| 0.89 \| \| 0.99 \| \| 3.21 \| \| 2.13 \| \| 0.90 \| \| 1.19 \| \| 1.18 \| \| 1.42 \| \| 2.04 \| \| 0.87 \| \| 1.37 \| \| 1.16 \| \| 1.23 \| \| 1.03 \| \| 0.87 \| \| 0.85 \| \| 4.11 \| \| 1.22 \| \| 0.95 \| \| 0.80 \| \| 1.62 \| \| 0.79 \| \| 36.00 \| \| 0.88 \| \| 1.36 \| \| 0.78 \| \| 1.03 \| \| 1.37 \| \| 1.05 \| \| 1.02 \| \| 2.04 \| \| 0.88 \| \| 1.20 \| \| 0.94 \| \| 1.87 \| \| 1.06 \| \| 1.25 \| \| 0.56 \| \| 0.95 \| \| 1.07 \| \| 1.56 \| \| 1.06 \| \| 1.74 \| \| 2.49 \| \| 0.98 \| \| 0.84 \| \| 0.82 \| \| 1.15 \| \| 1.24 \| \| 0.88 \| \| 1.21 \| \| 0.92 \| \| 0.99 \| \| 0.97 \| \| 1.02 \| \| 2.00 \| \| 1.04 \| \| 0.90 \| \| 1.04 \| \| 1.44 \| \| 1.27 \| \| 0.96 \| \| 1.05 \| \| 1.13 \| \| 1.17 \| \| 0.89 \| \| 1.10 \| \| 2.26 \| \| 1.02 \| \| 0.80 \| \| 1.14 \| \| 0.94 \| \| 0.79 \| \| 1.00 \| \| 1.22 \| | \| 0.44 \| \| --- \| \| 0.18 \| \| 0.22 \| \| 0.02 \| \| 0.05 \| \| 0.04 \| \| 0.92 \| \| 0.26 \| \| 0.05 \| \| 0.20 \| \| 0.57 \| \| 0.03 \| \| 0.06 \| \| 0.05 \| \| 0.08 \| \| 0.03 \| \| 0.10 \| \| 0.11 \| \| 0.05 \| \| 0.05 \| \| 0.07 \| \| 0.56 \| \| 0.08 \| \| 0.02 \| \| 0.03 \| \| 0.07 \| \| 0.08 \| \| 0.07 \| \| 0.44 \| \| 0.08 \| \| 0.16 \| \| 0.39 \| \| 0.07 \| \| 0.03 \| \| 0.57 \| \| 0.39 \| \| 0.65 \| \| 0.12 \| \| 0.56 \| \| 0.04 \| \| 0.08 \| \| 0.09 \| \| 0.05 \| \| 0.08 \| \| 0.09 \| \| 0.04 \| \| 0.14 \| \| 0.22 \| \| 0.04 \| \| 0.54 \| \| 0.42 \| \| 0.04 \| \| 0.02 \| \| 0.11 \| \| 0.04 \| \| 0.85 \| \| 0.69 \| \| 0.07 \| \| 0.21 \| \| 0.94 \| \| 0.11 \| \| 0.02 \| \| 0.89 \| \| 0.08 \| \| 0.06 \| \| 0.05 \| \| 0.03 \| \| 0.01 \| \| 0.26 \| \| 0.16 \| \| 0.03 \| \| 0.06 \| \| 0.05 \| \| 0.03 \| \| 0.01 \| \| 0.11 \| \| 0.39 \| \| 0.74 \| \| 0.02 \| \| 0.05 \| \| 0.07 \| \| 0.10 \| \| 0.21 \| \| 0.04 \| \| 0.05 \| \| 0.02 \| \| 0.05 \| \| 0.09 \| | \| 5.09 \| \| --- \| \| 0.85 \| \| 10.99 \| \| 2.19 \| \| 3.50 \| \| 3.39 \| \| 1.13 \| \| 1.88 \| \| 6.68 \| \| 1.51 \| \| 0.95 \| \| 3.86 \| \| 1.47 \| \| 7.91 \| \| 9.34 \| \| 4.73 \| \| 8.48 \| \| 3.28 \| \| 29.90 \| \| 5.93 \| \| 13.88 \| \| 1.16 \| \| 3.33 \| \| 4.08 \| \| 3.81 \| \| 4.88 \| \| 2.89 \| \| 2.47 \| \| 1.56 \| \| 5.42 \| \| 0.71 \| \| 0.95 \| \| 2.10 \| \| 32.73 \| \| 2.61 \| \| 0.70 \| \| 2.09 \| \| 2.14 \| \| 4.48 \| \| 2.87 \| \| 7.17 \| \| 3.32 \| \| 1.70 \| \| 9.48 \| \| 2.99 \| \| 11.69 \| \| 2.00 \| \| 4.70 \| \| 3.59 \| \| 2.16 \| \| 2.97 \| \| 1.99 \| \| 2.45 \| \| 0.27 \| \| 2.86 \| \| 21.56 \| \| 4.80 \| \| 6.52 \| \| 1.39 \| \| 1.10 \| \| 15.48 \| \| 3.16 \| \| 1.35 \| \| 3.57 \| \| 2.78 \| \| 3.23 \| \| 2.92 \| \| 3.09 \| \| 16.88 \| \| 2.76 \| \| 2.44 \| \| 1.86 \| \| 4.12 \| \| 5.83 \| \| 1.65 \| \| 2.87 \| \| 1.96 \| \| 8.99 \| \| 1.63 \| \| 3.93 \| \| 9.15 \| \| 2.19 \| \| 0.68 \| \| 2.61 \| \| 2.40 \| \| 1.63 \| \| 2.73 \| \| 3.01 \| |
|  | | | | | |

| **Supplementary Table 2. Classification of differentially expressed genes** | | | |
| --- | --- | --- | --- |
| GeneBank ID | Gene Symbol | Gene title | Fold Change |
| **TGF-beta/BMP signaling pathway associated genes** | | | |
| NM_007554 | *Bmp4* | Bone morphogenetic protein 4 | 6.68 |
| NM_009367 | *Tgfb2* | Transforming growth factor, beta 2 | 4.12 |
| NM_009368 | *Tgfb3* | Transforming growth factor, beta 3 | 5.83 |
| NM_009758 | *Bmpr1a* | Bone morphogenetic protein receptor, type 1A | 3.86 |
| NM_010754 | *Smad2* | MAD homolog 2 (Drosophila) | 3.23 |
| NM_016769 | *Smad3* | MAD homolog 3 (Drosophila) | 2.92 |
| NM_008540 | *Smad4* | MAD homolog 4 (Drosophila) | 3.09 |
| NM_009820 | *Runx2* | Runt related transcription factor 2 | 3.16 |
| NM_009931 | *Col4a1* | Collagen, type IV, alpha 1 | 4.08 |
| NM_010206 | *Fgfr1* | Fibroblast growth factor receptor 1 | 2.87 |
| NM_016779 | *Dmp1* | Dentin matrix protein 1 | 32.73 |
| **Hypertrophic marker genes** | | | |
| NM_009925 | *Col10a1* | Collagen, type X, alpha 1 | 4.73 |
| NM_007742 | *Col1a1* | Collagen, type I, alpha 1 | 5.93 |
| NM_009820 | *Runx2* | Runt related transcription factor 2 | 3.16 |
| NM_016779 | *Dmp1* | Dentin matrix protein 1 | 32.73 |
| **ECM molecular** | | | |
| NM_181277 | *Col14a1* | Collagen, type XIV, alpha 1 | 29.90 |
| NM_007742 | *Col1a1* | Collagen, type I, alpha 1 | 5.93 |
| NM_009925 | *Col10a1* | Collagen, type X, alpha 1 | 4.73 |
| NM_009931 | *Col4a1* | Collagen, type IV, alpha 1 | 4.08 |
| NM_007542 | *Bgn* | Biglycan | 3.50 |
| **ITG family** | | | |
| NM_010575 | *Itgα2b* | Integrin alpha 2b | 3.59 |
| NM_010578 | *Itgb1* | Integrin beta 1 (fibronectin receptor beta) | 2.45 |

Fold change column represents fold changes of each gene expression in Homo as compared with the expression in WT.

**Supplementary Table 3. Clinical information of OA patients**

|  |  | | Mankin score | |
| --- | --- | --- | --- | --- |
| Case | Sex | Age | Injured | Unaffected |
| 1 | Female | 68 | 2 | 11 |
| 2 | Female | 59 | 1 | 8 |
| 3 | Male | 71 | 1 | 9 |
| 4 | Female | 71 | 2 | 11 |
| 5 | Female | 76 | 1 | 10 |
| 6 | Female | 68 | 1 | 11 |
| 7 | Female | 74 | 2 | 10 |
| 8 | Male | 61 | 2 | 9 |
| 9 | Female | 63 | 1 | 9 |
| 10 | Male | 70 | 2 | 11 |

| **Supplementary Table 4. All primers for Real-time RT-PCR assay** | |
| --- | --- |
| Gene | Primer sequence |
| **Human** |  |
| *GAPDH* | Sense: 5’-AGAAAAACCTGCCAAATATGATGAC-3’;  Antisense: 5’-TGGGTGTCGCTGTTGAAGTC-3’; |
| *ID1* | Sense: 5’-CTGCTCTACGACATGAACGG-3’;  Antisense: 5’-GAAGGTCCCTGATGTAGTCGAT-3’; |
| *ID2* | Sense: 5’-AGTCCCGTGAGGTCCGTTAG-3’;  Antisense: 5’-AGTCGTTCATGTTGTATAGCAGG-3’; |
| *DLX5* | Sense: 5’-TTCCAAGCTCCGTTCCAGAC-3’;  Antisense: 5’-GAATCGGTAGCTGAAGACTCG-3’; |
| *RUNX2* | Sense: 5’-AGAAGGCACAGACAGAAGCTTGA-3’;  Antisense: 5’-AGGAATGCGCCCTAAATCACT-3’; |
| *MMP13* | Sense: 5’-ACTGAGAGGCTCCGAGAAATG-3’;  Antisense: 5’-GAACCCCGCATCTTGGCTT-3’; |
| *COL10A1* | Sense: 5’-ATGCTGCCACAAATACCCTTT-3’;  Antisense: 5’-GGTAGTGGGCCTTTTATGCCT-3’; |
| *COL2A1* | Sense: 5’-GGCAATAGCAGGTTCACGTACA-3’;  Antisense: 5’-CGATAACAGTCTTGCCCCACTT-3’; |
| *ACVR1* | Sense: 5’-GTGAAGGTCTCTCCTGCGGTA-3’;  Antisense: 5’-GCCATCGTTGATGCTCAGTGA-3’; |
| *STAT1* | Sense: 5’-CAGCTTGACTCAAAATTCCTGGA-3’;  Antisense: 5’-TGAAGATTACGCTTGCTTTTCCT-3’; |
| *SERPINE1* | Sense: 5’-ACCGCAACGTGGTTTTCTCA-3’;  Antisense: 5’-TTGAATCCCATAGCTGCTTGAAT-3’; |
| *SMAD2* | Sense: 5’-CGTCCATCTTGCCATTCACG-3’;  Antisense: 5’-CTCAAGCTCATCTAATCGTCCTG-3’; |
| *SMAD3* | Sense: 5’-TGGACGCAGGTTCTCCAAAC-3’;  Antisense: 5’-CCGGCTCGCAGTAGGTAAC-3’; |
| *MMP9* | Sense: 5’-TGTACCGCTATGGTTACACTCG-3’;  Antisense: 5’-GGCAGGGACAGTTGCTTCT-3’; |
| *ADAMTS4* | Sense: 5’-GAGGAGGAGATCGTGTTTCCA-3’;  Antisense: 5’-CCAGCTCTAGTAGCAGCGTC-3’; |
| *ADAMTS5* | Sense: 5’-GAACATCGACCAACTCTACTCCG-3’;  Antisense: 5’-CAATGCCCACCGAACCATCT-3’; |
| **Mouse** |  |
| *Gapdh* | Sense: 5’-AGGTCGGTGTGAACGGATTTG-3’;  Antisense: 5’-TGTAGACCATGTAGTTGAGGTCA-3’; |
| *Acvr1* | Sense: 5’-GTGGAAGATTACAAGCCACCA-3’;  Antisense: 5’-GGGTCTGAGAACCATCTGTTAGG-3’; |
| *Stat1* | Sense: 5’-TCACAGTGGTTCGAGCTTCAG-3’;  Antisense: 5’-GCAAACGAGACATCATAGGCA-3’; |
| *Serpine1* | Sense: 5’-TTCAGCCCTTGCTTGCCTC-3’;  Antisense: 5’-ACACTTTTACTCCGAAGTCGGT-3’; |
| *Id1* | Sense: 5’-CTGCTCTACGACATGAACGG-3’;  Antisense: 5’-GAAGGTCCCTGATGTAGTCGAT-3’; |
| *Id2* | Sense: 5’-AGTCCCGTGAGGTCCGTTAG-3’;  Antisense: 5’-AGTCGTTCATGTTGTATAGCAGG-3’; |
| *Dlx5* | Sense: 5’-TCTCTAGGACTGACGCAAACA-3’;  Antisense: 5’-GTTACACGCCATAGGGTCGC-3’; |
| *Col2a1* | Sense: 5’-GGGAATGTCCTCTGCGATGAC-3’;  Antisense: 5’-GAAGGGGATCTCGGGGTTG -3’; |
| *Runx2* | Sense: 5’-CCAACCGAGTCATTTAAGGCT-3’;  Antisense: 5’-GCTCACGTCGCTCATCTTG-3’; |
| *Col10a1* | Sense: 5’-TTCTGCTGCTAATGTTCTTGACC-3’;  Antisense: 5’-GGGATGAAGTATTGTGTCTTGGG-3’; |
| *Dmp1* | Sense: 5’-CTGAAGAGAGGACGGGTGATT-3’;  Antisense: 5’-CGTGTGGTCACTATTTGCCTG-3’. |

| **Supplementary Table 5. Concentration and RNA quality score (RQS) of RNA samples** | | | |
| --- | --- | --- | --- |
| Figure | RNA sample | Concentration (ng/μL) | RQS |
| Fig. 1a,1e, 3d, 3e & Supplementary Fig. 1a  Fig. 1g  Fig. 1i & Supplementary Fig. 1c  Fig. 2a, 3g  Fig. 2c & Supplementary Fig. 2f, 2o, 3b  Fig. 2f  Fig. 4g  Fig. 4h  Fig. 6f  Fig. 6g  Fig. 7c  Supplementary Fig. 2a  Supplementary Fig. 2c  Supplementary Fig. 2h, 2j, 3d  Supplementary Fig. 2n  Supplementary Fig. 6b, c | WT1  WT2  WT3  WT4  Hetero1  Hetero2  Hetero3  Hetero4  Homo1  Homo2  Homo3  Homo4  WT+Vehicle  Homo+Vehicle  Homo+COL2A1  WT NC  WT *Col2a1-*si1  WT *Col2a1-*si2  Homo NC  SW1353+Vehicle  SW1353+COL2A1  Hs819.T+Vehicle  Hs819.T+COL2A1  AC NC  AC *COL2A1-*si1  AC *COL2A1*-si2  CHO+Vehicle  Hyper+Vehicle  Hyper+COL2A1  SW1353 Vehicle+IgG  SW1353 Vehicle+ITGB1 blk ab  SW1353 COL2A1+IgG  SW1353 COL2A1+ITGB1 blk ab  Hs819.T Vehicle+IgG  Hs819.T Vehicle+ITGB1 blk ab  Hs819.T COL2A1+IgG  Hs819.T COL2A1+ITGB1 blk ab  SW1353 Vehicle1+Vehicle2  SW1353 U0126+Vehicle2  SW1353 COL2A1+Vehicle1  SW1353 COL2A1+U0126  Hs819.T Vehicle1+Vehicle2  Hs819.T U0126+Vehicle2  Hs819.T COL2A1+Vehicle1  Hs819.T COL2A1+U0126 Unaffected1  Unaffected2  Unaffected3  Unaffected4  Unaffected5  Unaffected6  Unaffected7  Unaffected8  Unaffected9  Unaffected10  Injured1  Injured2  Injured3  Injured4  Injured5  Injured6  Injured7  Injured8  Injured9  Injured10  Human articular chondrocyte  293T  SW1353  Hs819.T  Mouse articular chondrocyte  ATDC5  ATDC5 Scramble-sh  ATDC5 *Col2a1-*sh1  ATDC5 *Col2a1-*sh2  AC Vehicle  AC COL2A1  SW1353 Vehicle1+Vehicle2  SW1353 SP600125+Vehicle2  SW1353 COL2A1+Vehicle1  SW1353 COL2A1+SP600125  Hs819.T Vehicle1+Vehicle2  Hs819.T SP600125+Vehicle2  Hs819.T COL2A1+Vehicle1  Hs819.T COL2A1+SP600125 | 187.47  127.36  133.74  125.09  111.40  164.30  176.23  178.14  187.83  182.83  160.72  109.45  247.11  187.18  249.41  235.88  216.66  354.49  261.04  126.53  108.60  151.60  133.28  172.09  182.09  179.21  166.65  216.63  263.86  212.33  230.95  217.02  156.21  245.64  270.10  226.71  235.30  203.88  215.55  152.44  162.30  166.67  164.62  139.87  147.59  158.48  135.70  127.06  147.90  147.94  169.72  139.28  125.16  110.65  95.24  151.42  209.59  100.22  191.92  189.46  195.05  101.65  173.09  104.57  111.70  294.08  326.33  321.18  460.94  537.71  418.67  298.26  263.44  288.53  178.12  192.91  282.15  195.07  197.25  214.64  228.90  224.10  210.97  234.85 | 8.9  8.9  9.0  9.0  8.0  8.2  8.5  8.6  8.4  9.0  9.1  8.7  9.4  9.3  9.3  9.2  9.1  8.9  8.9  9.6  8.8  9.0  9.5  8.8  8.9  8.8  8.9  8.8  8.8  8.8  9.2  9.2  9.2  9.5  8.9  9.5  9.7  8.8  8.8  8.9  9.0  9.0  9.1  8.9  9.2  9.2  9.9  9.7  8.7  9.2  9.0  8.8  8.9  8.9  8.9  9.0  8.6  9.0  9.0  8.9  9.9  8.2  8.7  8.6  9.3  8.8  9.0  9.7  9.4  9.1  9.5  9.5  9.5  9.4  9.0  9.2  8.5  9.0  8.8  8.7  9.2  9.0  8.9  8.9 |
